# NESmapper: Accurate Prediction of Leucine-Rich Nuclear Export Signals Using Activity-Based Profiles

**DOI:** 10.1371/journal.pcbi.1003841

**Published:** 2014-09-18

**Authors:** Shunichi Kosugi, Hiroshi Yanagawa, Ryohei Terauchi, Satoshi Tabata

**Affiliations:** 1 Kazusa DNA Research Institute, Kisarazu, Chiba, Japan; 2 Department of Biosciences and Informatics, Faculty of Science and Technology, Keio University, Yokohama, Japan; 3 Iwate Biotechnology Research Center, Kitakami, Iwate, Japan; UCSD, United States of America

## Abstract

The nuclear export of proteins is regulated largely through the exportin/CRM1 pathway, which involves the specific recognition of leucine-rich nuclear export signals (NESs) in the cargo proteins, and modulates nuclear–cytoplasmic protein shuttling by antagonizing the nuclear import activity mediated by importins and the nuclear import signal (NLS). Although the prediction of NESs can help to define proteins that undergo regulated nuclear export, current methods of predicting NESs, including computational tools and consensus-sequence-based searches, have limited accuracy, especially in terms of their specificity. We found that each residue within an NES largely contributes independently and additively to the entire nuclear export activity. We created activity-based profiles of all classes of NESs with a comprehensive mutational analysis in mammalian cells. The profiles highlight a number of specific activity-affecting residues not only at the conserved hydrophobic positions but also in the linker and flanking regions. We then developed a computational tool, NESmapper, to predict NESs by using profiles that had been further optimized by training and combining the amino acid properties of the NES-flanking regions. This tool successfully reduced the considerable number of false positives, and the overall prediction accuracy was higher than that of other methods, including NESsential and Wregex. This profile-based prediction strategy is a reliable way to identify functional protein motifs. NESmapper is available at http://sourceforge.net/projects/nesmapper.

This is a *PLOS Computational Biology* Software Article

## Introduction

The nuclear export of proteins controls their nuclear or cytoplasmic functions in response to physiological conditions, including the cell cycle or extracellular stimuli, and antagonizes the nuclear import activities mediated by the importin family. Nuclear export is mediated by the interaction of nuclear export signals (NESs) with exportin/CRM1 or Msn5p in yeast, members of the importin beta family. The CRM1–Ran–GTP complex binds directly to the NES in the cargo protein and directs the export of the ternary complex from the nucleus. The cargo is released from the complex by the hydrolysis of Ran–GTP to Ran–GDP in the cytoplasm [1]–[3]. Over 200 NESs have been identified experimentally and their dependence on CRM1 has been confirmed using leptomycin B (LMB), a specific inhibitor of CRM1, which binds covalently to the cysteine residue of CRM1 [4]. Eighty-four percent of identified NESs are LMB-sensitive NESs [5] and the subcellular localizations of 285 proteins in fission yeast [6] and >100 proteins in HeLa [7] cells were altered after treatment with LMB, indicating that CRM1 constitutes the major nuclear export pathway. Moreover, CRM1 is a potential therapeutic target because the nuclear export of many tumor-associated proteins has been deregulated in various cancers [8], [9].

The CRM1-dependent NESs typically contain conserved large hydrophobic residues, with several patterns of spacing. The proposed consensus sequence, designated the “classical consensus”, is Φ–X2,3–Φ–X2,3–Φ–X–Φ, where Φ represents L, I, V, M, or F and X2,3 any two or three amino acids [10]–[13]. This consensus sequence fits ∼70% of the experimentally defined NESs but is frequently found in many proteins that are not transported by CRM1. Our previous study using a newly developed NES screening system with artificial peptide libraries identified two new classes, class 2 (Φ–X–Φ–X2–Φ–X–Φ) and class 3 (Φ–X2–Φ–X3–Φ–X2–Φ), in addition to the classical class 1 [14]. The class 1 NES contains subclasses 1a (Φ–X3–Φ–X2–Φ–X–Φ), 1b (Φ–X2–Φ–X2–Φ–X–Φ), 1c (Φ–X3–Φ–X3–Φ–X–Φ), and 1d (Φ–X2–Φ–X3–Φ–X–Φ). More strict consensus sequences proposed are Φ–X1,2–[∧P]– Φ–[∧P]2,3–Φ–[∧P]– Φ for class 1, Φ–[∧P]– Φ–[∧P]2–Φ–[∧P]– Φ for class 2, and Φ–X–[∧P]– Φ–[∧P]3–Φ–[∧P]2–Φ for class 3, where [∧P]2,3 represents any two or three amino acids except proline and C, W, A, or T are permitted only at one of the four conserved hydrophobic positions. Because a stretch of hydrophobic residues as well as proline in the spacer regions has an inhibitory effect on the NES function, these consensus sequences do not include hydrophobic stretches with more than four consecutive hydrophobic residues overlapping the second and third conserved hydrophobic residues [14]. A recent bioinformatic analysis of NES sequences and structures using a newly generated NES database proposed refined consensus patterns based on our consensus sequences, where neither C, W, A, nor T is permitted at C-terminal hydrophobic positions Φ3 or Φ4 [15]. Using structural analyses for CRM1-NES complexes, Güttler et al (2010) demonstrated that CRM1 has five pockets for binding the conserved hydrophobic residues of NES and that one more hydrophobic position can be extended to the N-terminus of the class 1a NES consensus, represented as Φ–X2–Φ–X3–Φ–X2–Φ–X–Φ.

The crystal structures of CRM1–NES complexes reveal a narrow and rigid conformation of the CRM1-binding grooves of the NES hydrophobic cores, whereas the NESs adopt relatively flexible structures to bind CRM1 [16]–[19]. The CRM1-binding conformation of the prototypic PKI NES is an α-helical structure, whereas the HIV-1 NES binds in an extended loop conformation [16]. This structural flexibility of NES binding explains the different spacings of the NES hydrophobic positions. A bioinformatic analysis of the structures of the NES-containing proteins in the Protein Data Bank demonstrated that NESs tend to be exposed on the protein surfaces and form an α-helical conformation in the N-terminal regions and a loop conformation at the C-terminus whereas nonfunctional NESs tend to form an α-helix in the entire regions [15]. However, that study suggested that the consensus-sequence-based prediction of NESs is difficult to achieve with improved accuracy even when predictions of their secondary structures and protein surface exposure are incorporated into currently available prediction tools [15].

NES prediction from NES consensus sequences produces a great number of sequences that do not function as NESs, mainly because of the nature of hydrophobic-residue-rich sequences, which are frequently present in the internal hydrophobic regions of modular proteins or membrane-anchoring domains. Three computational methods for NES prediction have been reported that do not depend on consensus sequences alone. la Cour et al. (2004) first reported that NESs are located in flexible, surface-accessible regions and form α-helical structures in proteins. They also found that the non-hydrophobic regions of NESs are enriched in acidic residues. They developed the first NES prediction tool, NetNES, using a machine learning approach combining neural networks and hidden Markov models with NESs (NESbase) collected from the literature [20]. Another NES predictor, NESsential, uses the meta-features of NESs, including their disordered structure and solvent accessibility (predicted computationally) combined with trained modeling with a support vector machine [21]. It has been shown that the disordered features around NESs can effectively discriminate functional NESs from false positives, and NESsential shows better prediction accuracy than NetNES [21]. Short linear motifs have been shown to be preferentially located in the intrinsically disordered regions of proteins, allowing flexible and easily accessible interactions with their motif-interactors [22]. The observation that NESs are preferentially present in disordered regions suggests that the NES functions, at least in part, as a linear motif, such as the nuclear localization signal (NLS). The other recently developed tool, Wregex [23], predicts linear protein motifs including NESs using an approach similar to MEME [24] and Scansite [25], which use position-specific scoring matrices (PSSMs) for motif prediction. In Wregex, the PSSMs of NESs have been created with experimentally verified NESs, including those from the ValidNES database [5] and the human deubiquitinase family [26].

In our previous study, we demonstrated that each amino acid residue comprising the classical NLSs contributes independently and additively to the entire NLS activity, and that the strength level of the NLS activity can be predicted using its activity-based profile generated with mutational assays of NLS activity [27]. In this study, we applied this method to NES prediction, combined with scores that were calculated from the features of the amino acid composition outsides the NES. We show that this approach more accurately predicts NESs than other current methods.

## Design and Implementation

### NES data sets

**Figure 1:**
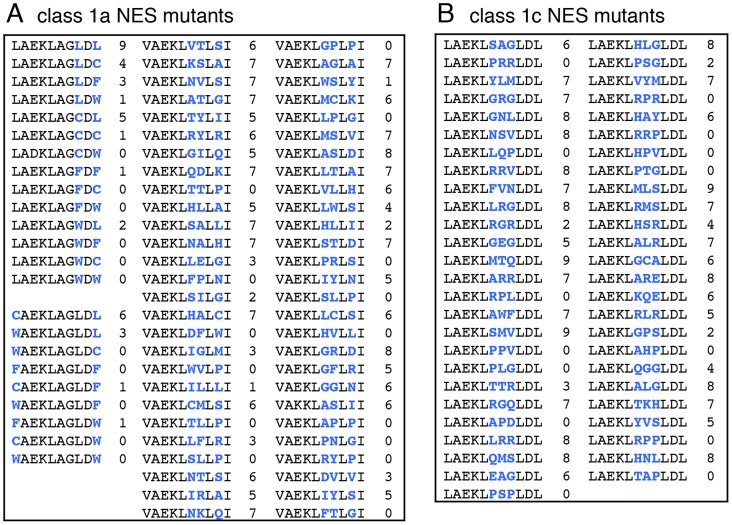
Nuclear export activity of class 1a and class 1c NES mutants. (**A**) Class 1a NESs carrying mutations at two hydrophobic positions and three spacer positions between Φ2 and Φ4. (**B**) Class 1c NESs carrying mutations at three positions within the spacer region between Φ2 and Φ3. These NES mutants were assayed for their nuclear export activity in NIH3T3 cells, and the activities were classified as scores from 1 to 10, as in Figure S1. The scores are indicated at the right columns of the corresponding sequences. Altered bases are highlighted in blue.

**Figure 2:**
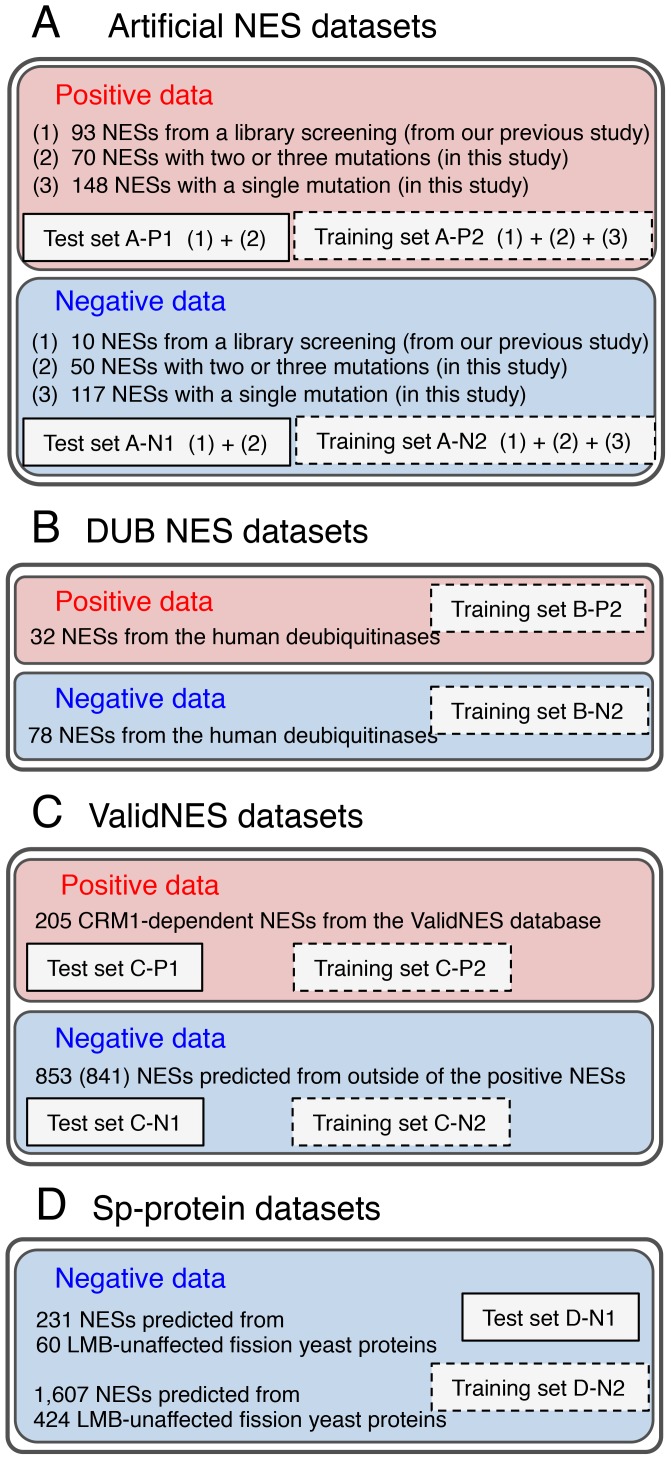
Positive and negative NES datasets obtained from four different data resources. (**A**) Artificial NES datasets. (**B**) DUB NES datasets. (**C**) Valid NES datasets. (**D**) Sp-protein datasets. The positive and negative datasets (B-P2 and B-N2) of the DUB datasets and the negative training dataset (D-N2) of the Sp-protein datasets were always included in the training data for the profile optimization, whereas the other training datasets were used only when they were not contained in a test dataset to be used. For example, when we conducted the prediction test with the test datasets, A-P1 and A-N1, we used the optimized profiles for NESmapper, that were trained with C-N2, in addition to B-P2, B-N2, and D-N2.

We used three positive NES sets, consisting of 205 NESs from the ValidNES database (ValidNES dataset) [5], 32 NESs from the DUB NES dataset [26], and 311 artificial NESs from our studies (Table S1, Figure 1) (positive artificial NES dataset), including 93 NESs obtained in a previous study by library screening [14]. For generating training datasets to optimize NES profiles, we prepared four negative NES datasets consisted of 1,607 potentially nonfunctional NESs predicted from 424 LMB-unaffected fission yeast proteins (Sp-proteins) [6], 853 potentially nonfunctional NESs predicted from regions other than confirmed NES positions in the positive ValidNES dataset, 78 NESs from the DUB NES dataset, and 177 artificial NESs from our studies (Table S1, Figure 1) (negative artificial NES dataset). Detailed descriptions are provided in Text S1 in Supporting Information, and the constitution of the datasets used in this study is schematically represented in Figure 2.

### Measurement of nuclear export activities and generation of NES profiles

Double-stranded oligonucleotides encoding NES variants were inserted into the *XbaI* and *BamHI* sites of pCMV-GFP, as described previously [14]. Plasmid clones encoding NESs containing ∼19 different amino acid at each position within an NES template were selected from ∼48 randomly selected bacterial colonies. The template NES sequences for five NES classes were designed based on the prototypical NES of cyclic AMP-dependent protein kinase inhibitor (PKI NES) [28], and were LMB-sensitive. The mouse fibroblast NIH3T3 cell line was transfected with the plasmids (∼1.0 µg each) using 2 µl of jet-PEI (PolyPlus-transfection, Strasbourg, France) as described previously [29], and the green fluorescent protein (GFP) fluorescence was observed after culture for 36–48 h. The nuclear export activities of the NESs were measured semi-quantitatively according to the observed GFP localization phenotypes, as shown in Figure S1. An NES profile for each subclass was generated from the determined NES scores. Blanks in the NES profiles that remained undetermined were filled with scores postulated from the amino acid similarities or profiles of different NES classes.

### Optimization of NES profiles by training

To allow the faithful calculation of the NES activities, the scores in the NES profiles were optimized to fit the calculation for NESmapper by computational training with positive and negative NES training datasets. Detailed descriptions are provided in Text S1.

### Amino acid properties in regions flanking NESs

Short linear motifs tend to occur in intrinsically disordered regions [22]. Although many NESs are also located in disordered regions, a significant number of NESs are likely to be located in ordered regions [15], [21]. We computed the amino acid compositions of the flanking regions of positive and negative NESs. The positive dataset consisted of 178 LMB-sensitive NESs from the ValidNES dataset, and the negative datasets of 1,259 potentially nonfunctional NESs from the ValidNES dataset and 2,078 NESs from the Sp-protein dataset. Only NESs that had at least 25 amino acid residues at both the flanking sides were selected. The 25-amino-acid flanking regions, especially the N-terminal flanking regions, of positive NESs had few hydrophobic amino acids and were richer in polar amino acids and proline than were negative NESs (Figure S2A–D). The C-terminal flanking regions of the positive NESs were also richer in acidic but not basic amino acids than those of the negative NESs (Figure S2E–H). We created frequency distribution tables of a hydrophobic-to-polar amino acid ratio (HPR) in the 25-amino-acid N-terminal flanking regions and the net charge (NC) of the 25-amino-acid C-terminal flanking regions of NESs for the positive and negative NES datasets. We conducted the Fisher's exact test for the frequencies of HPR and NC for the positive and negative NES datasets. The test gave a p-value<0.0001 for the frequencies of the HPR categorized into ≤−2 and >2, and a p-value 0.034 for the frequencies of the NC categorized into ≤–2 and >2. Then, we calculated the likelihood ratios for each HPR and NC value (Tables S2 and S3). The likelihood ratio was decreased linearly as HPR increased, with a threefold change in the ValidNES dataset and an over 10-fold change in the ValidNES/Sp-protein dataset (Table S3). The likelihood ratios for NC exhibited a similar distribution, with changes of about twofold for both the datasets (Table S3). This observation suggests that the properties of the amino acids composing the NES-flanking regions can be one of the classifiers that discriminate true from false NESs in proteins.

### Calculation of nuclear export activities of NESs in proteins with NESmapper

The NES scores were calculated using the NES profiles, as described previously [27], but a manual score adjustment procedure based on experiments with a GFP reporter carrying double motifs was replaced with a computational profile-optimization method, as described in the previous section. To calculate the activity score (*Ts*) for an NES, the standard score of the template NES sequence used to generate the profile was subtracted from the scores in the profiles corresponding to each position and residue of the NES. The subtracted scores were summed and the standard score was then added to the summed score. The above calculation is shown by the following equation.
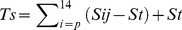
where *Sij* is the score corresponding to position *i* and amino acid *j* in the profile, *St* is the standard score, and *p* is the start position of the profile (i.e., *p* = 1–4, depending on the window position on the query sequence). To reduce false NESs that overlap with the hydrophobic regions in the proteins, such as membrane-spanning regions and regions embedded inside the protein, a hydrophobicity rate (content of hydrophobic residues) in the spacer regions of an NES was calculated and a penalty score (i.e., −7, which was based on the observation that the activity of a class 1a NES with score 8 was decreased in a level of score 1 when three spacer residues were converted to hydrophobic residues) was added to the total score for an NES with a hydrophobicity rate ≥0.4. This function reduced false positives by 13% in an NES dataset from the ValidNES database. The NESmapper program scans the protein sequence with a window size of 14 amino acid residues (11–13 amino acid residues in the N-terminal region) and a shift size of one amino acid, and finds NES sequences with a significant level of scores, which are calculated based on the NES profiles for class 1b, class 1c, class 2, and extended class 1a. Because the class 1d NESs constitute only a minor proportion of the ValidNES database and screened artificial NESs, we excluded the class 1d profile from the calculation to prevent an increase in false positives.

NESmapper also calculates the HPR of the N-terminal 25-amino-acid sequence flanking a predicted NES and the NC of the 25-amino-acid C-terminal flanking sequence, as described in the previous section. The NES score is multiplied by the predetermined likelihood ratios (2.5 for HPR≤30, 2 for HPR = 31–40, 1.4 for HPR = 41–50, 1 for HPR = 51–60, 0.6 for HPR = 61–80, 0.5 for HPR>80, 1.8 for ≦NC≤−4, and 0.6 for NC>0) corresponding to the calculated HPR and NC values, shown in Tables S2 and S3. This incorporation resulted in a slight reduction in the predicted false negatives or false positives, depending on the threshold score, and produced a robust prediction that was less affected by the threshold score (Table S4).

### Evaluation of NES prediction accuracy

Different positive and negative NES test sets were used to evaluate the prediction of NESs by NESmapper, NESsential, Wregex, NetNES, and NES consensus sequences. We used the same test datasets for the evaluation for each method, and designed several evaluation experiments with different test datasets. Detailed descriptions are provided in Text S1.

## Results

### Creation of activity-based NES profiles by mutational analysis

The relative activity of a motif can be calculated by adding the contribution of the corresponding amino acid at every position represented in an activity-based matrix profile of the motif, if the effects of the amino acids within the motif on the entire activity are independent and additive [27]. We investigated whether there are nonlinear correlations between the conserved hydrophobic residues within NESs using positive and negative NES datasets (see Text S1 for the datasets). The calculated frequencies of the amino acid occurrences at the conserved hydrophobic positions of the positive dataset of class 1 NES sequences (Table S5) were similar to those observed previously [13], [15]. The frequency of occurrence of an amino acid pair at two different positions (e.g., Val and Leu at Φ1 and Φ3) is expected to be a multiple of the frequencies at the two positions if the two amino acids do not interact specifically during the formation or function of the NES. In the negative dataset, every combination of two amino acids at the conserved positions correlated with the expected values (Table S6). In contrast, in the positive dataset, there were several patterns of hydrophobic pairs whose frequencies did not correlate with the expected values (i.e., <0.77-fold or >1.3-fold of the expected values, which gave a p-value = 0.0063 for the Fisher's exact test), indicating the presence of non-independent amino acid pairs at the conserved positions. However, the frequency of the non-independent pairs was relatively low (approximately 15% of all the observed frequencies of hydrophobic pairs) and the difference between the observed and expected frequencies was small (Table S6), which suggests that many of the amino acids, at least at hydrophobic positions, within the NES contribute independently to the entire activity of the NES.

**Figure 3:**
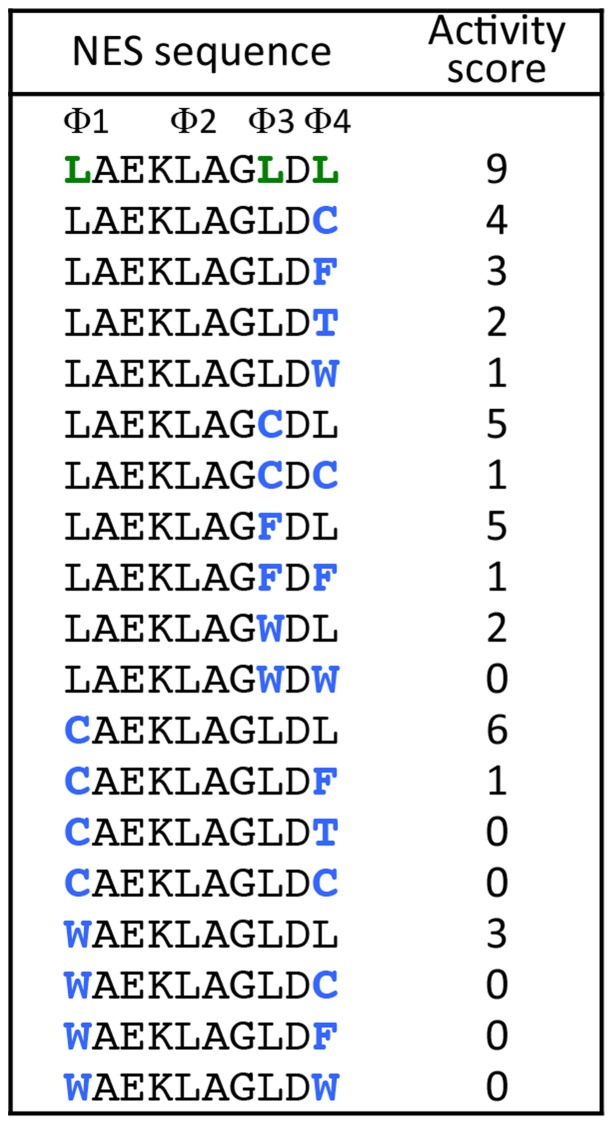
Independent and additive contributions of amino acids at the conserved hydrophobic positions to the entire NES activity. One or two leucine residues of a class 1a NES at the Φ1, Φ3, or Φ4 conserved hydrophobic positions, indicated on the top line, were replaced with cysteine, phenylalanine, threonine or tryptophan, as highlighted in blue, and the nuclear export activity was assayed in NIH3T3 cells. The indicated activity scores were determined as in Figure S1. Note that the effects of the substituted residues on the NES activity scores were roughly independent and additive.

**Figure 4:**
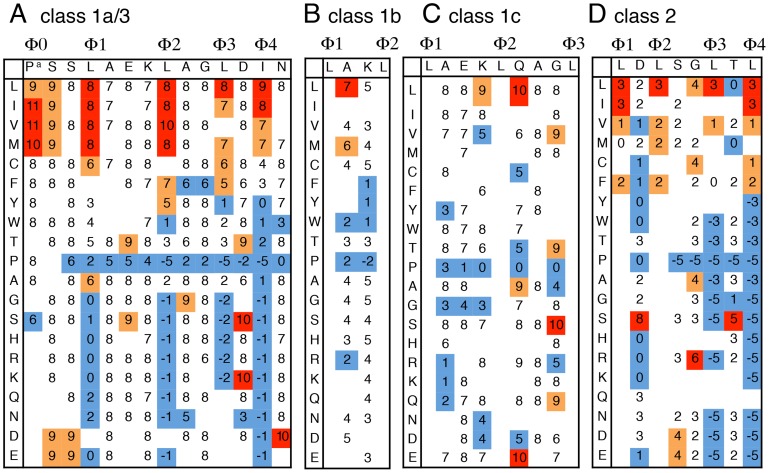
Activity-based profiles of CRM1-dependent NES. (**A**) Activity-based profile of class 1a/3 NES. Class 1a/3 NES is an extension of class 1a NES, in which the N-terminal region of class 1a and the C-terminal region of class 3 overlap. A single amino acid residue of a class 1a/3 NES template sequence, indicated at the top of the matrix, was replaced with the various other residues indicated in the left column. The nuclear export activity of the NES mutant was assayed in NIH3T3 cells. The indicated activity scores were determined as in Figure S1. This template NES has an activity score of 8. Scores with higher, slightly higher, and lower activities than the average value for each position are shown in red, orange, and blue, respectively. At several mutational positions, modified templates with a different level of basal activity were used to obtain more dispersed scores. The conserved hydrophobic positions (Φ0–Φ4) are marked on the template sequence. The scores at the Φ0 position (P^a^) were estimated based on the data of Güttler et al [16]. Blanks represent undetermined scores. (**B**) NES profile of the spacer region between the Φ1 and Φ2 positions of a class 1b NES. The template sequence has a standard activity score of 4. (PSSELAKLAGLDLN) (**C**) NES profile for the spacer regions between Φ1 and Φ3 positions of the class 1c NES. The template sequence (SELAEKLQAGLDLN) has an activity score of 8. (**D**) Activity-based profile of class 2 NESs. The template NES sequence, indicated at the top of the matrix, has a standard activity score of 3.

The independence of amino acids within an NES was also supported by a mutational analysis of the class 1a NESs. This analysis showed that many of the position-specific amino acids within an NES independently and additively contribute to the entire NES activity (Figure 3). We then attempted to create activity-based profiles for each NES class, as previously conducted for the classical NLS [27]. For the class 1a NESs, we prepared a modified sequence of the PKI NES for each NES class as a template and all the amino acid residues of the template NES were serially replaced with ∼20 other amino acid residues. The relative nuclear export activities of these altered sequences (a total of 791 sequence) were assayed in NIH3T3 cells and ranked from 1 to 10 based on the localization phenotype of the GFP reporter (see Figure S1 for details). The template NESs were LMB-sensitive and had a similar NES activity in yeast, suggesting that the assayed NES variants are CRM1-dependent NESs that function in diverse eukaryotic species. The profiles of the five subclasses of NESs were represented as scoring matrices based on their relative NES activities (Figure 4). A consensus sequence (Φ–X2–Φ-X3–Φ–X2–Φ–X–Φ) proposed by Güttler et al. [16] has one additional hydrophobic position at the N-terminal Φ0. We found that the N-terminal part of this consensus sequence matches the class 3 consensus, indicating that Güttler's consensus sequence represents a fusion of the class 3 and class 1a NESs. Therefore, we generated a profile for an extended class 1a corresponding to Güttler's consensus sequence by merging the results of the mutational assays of the class 3 NES with the class 1a profile (Figure 4A). The profiles show that different amino acids in the spacer regions, as well as those in the hydrophobic positions, contribute to the NES activity to different extents, depending on their positions. Proline functioned as a strong repressor in the entire spacer region, including the C-terminal flanking position, and this effect became stronger toward the C-terminal end. Acidic amino acids, asparagine and tryptophan, in the spacer regions act as position-dependent repressors. Leucine and isoleucine at conserved positions had a similarly strong effect on the NES activity, and cysteine, alanine, threonine, and tryptophan also made positive but weak contributions. The NES profiles suggest that combinations of amino acids with different levels of activity-directed effects generate various patterns of NESs.

### NES prediction performance of NESmapper with unoptimized and optimized NES profiles

We developed an NES prediction program, NESmapper, that calculates an NES score using the activity-based profiles for the class 1b, 1c, and 2, and the extended class 1a NESs. The performance of NESmapper with optimized NES profiles by training and unoptimized ones was evaluated using experimentally verified artificial NES test sets comprising 163 positive and 60 negative NESs (Table 1). NESmapper predictions with unoptimized profiles gave a sensitivity of 0.96 and a specificity of 0.85 for a threshold score of 2. Predictions with optimized profiles reduced the false positives for any threshold score, whereas predictions with profiles optimized with datasets excluding the test sets increased the false negatives. We used another test set, ValidNES-test, which contains 92 proteins (100 NESs) randomly selected from the ValidNES dataset. We regarded as false positives potentially nonfunctional NESs called from regions other than the ranges corresponding to the true NESs of the ValidNES-test set. For this test set, NESmapper with unoptimized profiles called 74% of true NESs for a threshold score of 2, and the predictions with the optimized profiles reduced the false positives by 40%–68% relative to those with the unoptimized profiles, at the expense of a slight increase in false negatives (Table 1). For another negative test set (Sp-test), which contained 60 proteins randomly selected from the Sp-protein dataset, predictions with the optimized profiles reduced the calls of potentially nonfunctional NESs (i.e., false positives) by approximately 60% relative to those with unoptimized profiles (Table 1). These results indicate that the optimization of NES profiles by training significantly reduced the number of false positives.

**Table 1:** Improved performance of NESmapper by optimization the profiles.

Test data^a^	Threshold score	NES prediction accuracy					
		Unoptimized profiles		Optimized profiles^b^		Optimized profiles^c^	
		False negatives (Sensitivity)	False positives (Specificity)	False negatives (Sensitivity)	False positives (Specificity)	False negatives (Sensitivity)	False positives (Specificity)
(1) Artificial NES sets	1	5 (0.969)	20 (0.667)	13 (0.920)	13 (0.783)	6 (0.963)	11 (0.817)
	2	6 (0.963)	9 (0.850)	19 (0.883)	6 (0.900)	6 (0.963)	6 (0.900)
	3	8 (0.951)	7 (0.883)	38 (0.767)	5 (0.917)	21 (0.871)	6 (0.900)
(2) ValidNES-test	1	21 (0.79)	587	28 (0.72)	346	25 (0.75)	276
	2	25 (0.75)	514	34 (0.66)	282	27 (0.73)	215
	3	25 (0.75)	447	37 (0.63)	212	37 (0.63)	177
(3) Sp-test	1	N.T.	235	N.T.	101	N.T.	95
	2	N.T.	199	N.T.	84	N.T.	76
	3	N.T.	160	N.T.	63	N.T.	55

a Three test datasets were used for the performance evaluation. (1) The artificial NES set: 163 positive and 60 negative experimentally verified NESs; (2) the ValidNES-test set containing 92 proteins (100 NESs) from the ValidNES database of positive and negative data; (3) the Sp-test set, a negative test set containing 50 proteins from the Sp-protein dataset.

b Profiles optimization was conducted using training data sets, excluding the corresponding test data.

c Profiles optimization was conducted using training data sets, including the corresponding test data.

N.T.: not tested.

### Comparison of prediction performance with other methods

We then compared the prediction performance of NESmapper using optimized and unoptimized NES profiles with the performances of predictions made with the traditional consensus sequence, the improved consensus sequences [14], [15], NetNES [13], Wregex [23], and NESsential [21]. We used the artificial NES test sets as the first test set, and the NES sequences were fused to the C-terminus of GFP for a fair evaluation of NetNES and NESsential, since NES peptides fused to the C-terminus of GFP are functional in our NES-assay system with mammalian cells and yeast. NESmapper and NESsential performed better, with higher sensitivities, than NetNES, Wregex, or the consensus-based methods, but NESmapper predicted a significantly lower number of false positives than NESsential (Table 2). As the second test sets, we used the ValidNES dataset containing 180 distinct proteins (205 NESs) for positive and negative data, although NESsential and Wregex have been developed using a subset from the ValidNES database. For another negative data, we used the Sp-test set, containing 60 proteins. The results with the second test sets indicated that the improved consensus sequences and NESsential (probability score ≥0.1) gave the best predictive performance in terms of sensitivity, which were approximately 0.05 or 0.1 higher than the sensitivity of NESmapper (score 2) (Table 3). However, of these five methods, NESmapper with optimized profiles predicted the lowest number of false positives: 16%–45% of the false positives predicted with the other methods (Table 3). For evaluation at a protein-level, NESmapper with optimized profiles predicted the lowest number of false positives for Sp-test set. Of the five methods, Wregex with the recommended configuration predicted the lowest number of false positives, but it displayed the highest number of false negatives (the lowest sensitivity) while using the PSSM that was created and trained with NESs from the ValidNES database. Current NES prediction methods, including NESmapper, still predict many false positives when predicting NESs from protein sequences. When we conducted an NES prediction analysis for 500 proteins randomly selected from the budding yeast protein database, these methods predicted 70∼98% of NES-containing proteins (Table S7). Although NESmapper predicted a lower number of false positives than other tools, NESmapper, as well as the other methods, may be more suitable for selecting candidate NESs from a protein set of interest rather than directly predicting CRM1-dependent nuclear export proteins from a proteome set.

**Table 2:** Prediction accuracies of NetNES, Wregex, NESsential, NESmapper, and consensus-based NES predictions using artificial NES test data.

Prediction method	Parameters	NES prediction accuracy	
		False negatives (Sensitivity)	False positives (Specificity)
Traditional consensus^a^	–	21 (0.871)	38 (0.367)
Improved consensus^b^	–	21 (0.871)	9 (0.850)
NetNES	–	47 (0.712)	22 (0.633)
Wregex	recommended config^c^	150 (0.080)	0 (1.000)
	relaxed config^d^	9 (0.945)	19 (0.683)
NESsential	p≥0.1^e^	8 (0.951)	42 (0.300)
	p≥0.5^f^	54 (0.669)	26 (0.567)
NESmapper	unoptimized profile^g^	6 (0.963)	9 (0.850)
	optimized profile^h^	19 (0.883)	6 (0.900)

Prediction accuracies of the indicated methods and tools were determined with the artificial NES sets, as in Table 1.

a Traditional NES consensus sequence, Φ–X2,3–Φ–X2,3–Φ–X–Φ.

b Class 1a, 1b, 1c, 1d, 2, and 3 NES consensus sequences, not allowing A, C, T, or W at positions Φ3 and Φ4 (see Introduction for detail).

c Prediction with recommended PSSM configuration.

d Prediction with relaxed PSSM configuration.

e NESs with the probability values of ≥0.1 selected.

f NESs with the probability values of ≥0.5 selected.

g Prediction with unoptimized NES profiles, NESs with a score of ≥2 were selected.

h Prediction with optimized NES profiles. NESs with a score of ≥2 were selected.

**Table 3:** Prediction accuracies of NetNES, Wregex, NESsential, NESmapper, and consensus-based NES predictions using the ValidNES/SpNES test data.

Prediction method	Parameters	NES prediction accuracy		
		ValidNES (185 proteins)		Sp-test (60 proteins)
		False negatives (Sensitivity)	False positives	False positives^e^
Traditional consensus	–	60 (0.707)	841	231 (93%)
Improved consensus	–	39 (0.810)	1,383	351 (98%)
NetNES	–	110 (0.463)	193	51 (78%)
Wregex	recommended config^a^	146 (0.288)	165	38 (50%)
	relaxed config^b^	43 (0.790)	1,791	487 (98.3%)
NESsential	p≥0.1^c^	38 (0.815)	783	166 (95%)
	p≥0.5^d^	163 (0.205)	35	8 (16%)
NESmapper	unoptimized, score ≥2	48 (0.766)	902	199 (93%)
	unoptimized, score ≥4	55 (0.732)	643	121 (85%)
	unoptimized, score ≥6	69 (0.663)	458	81 (72%)
	optimized, score ≥2	58 (0.717)	351	76 (70%)
	optimized, score ≥4	73 (0.644)	270	45 (50%)
	optimized, score ≥6	88 (0.571)	178	31 (40%)

Prediction accuracies were determined with the ValidNES dataset consisting of 185 proteins containing 205 LMB-sensitive NESs, as positive and negative data and the Sp-test negative dataset, containing 60 proteins from the Sp-protein dataset, as in Tables 1 and 2.

a Prediction with recommended PSSM configuration.

b Prediction with relaxed PSSM configuration.

c NESs with the probability values of ≥0.1 selected.

d NESs with the probability values of ≥0.5 selected.

e Percentage of proteins containing predicted NESs is indicated with parentheses.

**Figure 5:**
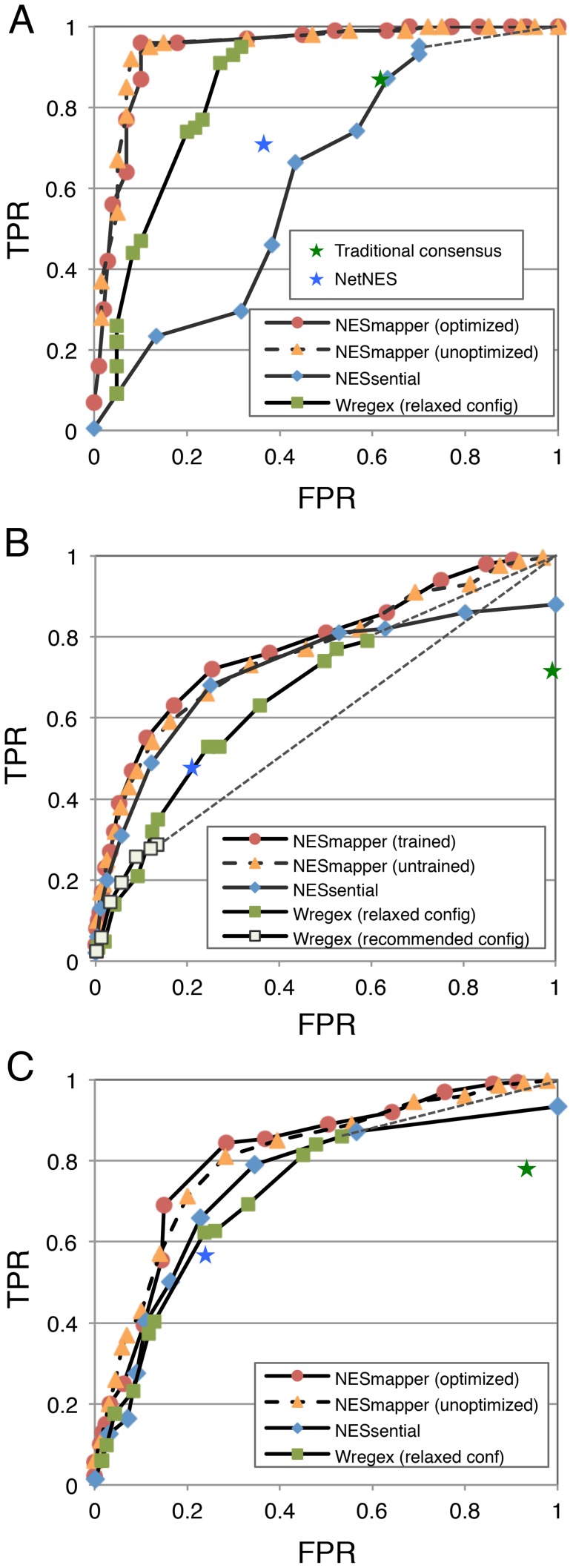
ROC analyses for five NES prediction methods. (**A**) ROC curve generated with artificial NES datasets. For the artificial NES sets, 163 positive and 60 negative experimentally verified NESs were used to plot the ROC curves for the traditional consensus-based prediction, NetNES, NESmapper, Wregex, and NESsential. The true positive rates (TPRs) and false positive rates (FPRs) for each tool were measured by changing the threshold scores for Wregex and NESmapper or the threshold probability values for NESsential. The curves for the NESmapper predictions with the optimized and unoptimized profiles are shown with solid lines with red circles and with dotted lines with orange triangles, respectively, those for Wregex with solid lines with green squares, and those for NESsential with solid lines with blue diamonds. The results for the traditional consensus-based prediction and NetNES are shown with green and blue asterisks, respectively. (**B**) ROC curve generated with ValidNES/Sp-test datasets. We measured the false positives by counting NESs called from regions other than the ranges corresponding to true NESs. To calculate the FPRs for the ValidNES and Sp-test datasets, only called NESs that matched the traditional consensus sequence were counted as false positives and divided by the number of sequences that matched the traditional consensus sequence in each dataset (841 for ValidNES and 231 for Sp-test). The mean FPRs for both datasets were used for the analysis. (**C**) ROC curve generated with the artificial NES and ValidNES/Sp-test datasets.

We then compared the performances of these methods, by plotting the receiver operating characteristic (ROC) curves and measuring the areas under the curves (AUCs) using two different sets of test NESs, the artificial NES and ValidNES/Sp-test datasets (Figure 5). For Wregex, only the data obtained with the relaxed configuration was used for the ROC analysis with the artificial NES datasets because the false negatives obtained with the recommended configuration were too high, as shown in Tables 2. With the artificial test datasets, the performance of NESmapper with both optimized profiles (AUC: 0.95) and unoptimized profiles (AUC: 0.94) was significantly better than that of other methods, the traditional consensus sequence, NetNES, Wregex (AUC: 0.85) and NESsential (AUC: 0.62). For also the ValidNES/Sp-test dataset, the performance of NESmapper (AUC: 0.78 and 0.75 for optimized and unoptimized profiles, respectively) was better than that of other methods, Wregex (AUC: 0.60) and NESsential (AUC: 0.72). The ROC analysis with combined datasets of the artificial and ValidNES/Sp-test datasets also showed that the performance of NESmapper with both optimized profiles (AUC: 0.81) and unoptimized profiles (AUC: 0.80) was better than that of other methods, Wregex (AUC: 0.73) and NESsential (AUC: 0.75). These results indicate that NESmapper can predict NESs more accurately than other NES prediction methods.

Another advantage of NESmapper was its running time. When an NES search against a set of 200 proteins with each 800 amino acid length was conducted, NESmapper took only eight seconds, whereas NESsential took over six hours through two steps of the sequential processes accompanying SABLE and POODLE-L (Table S8). Moreover, NESsential has a difficulty in treating sequences of large proteins, because POODLE-L accepts only proteins with <1000 amino acids.

### Conclusion

This study reveals the functional contributions of different amino acids at each position within and flanking an NES class, and demonstrates that each residue within an NES makes a largely independent and additive contribution to the entire nuclear export activity. Our NES prediction method based on activity-based profiles predicts NESs more accurately than other currently available methods, which is prominent especially in the context of linear peptide. Moreover, the fact that the performance of NESmapper is considerably better than that of Wregex suggests that the activity-based profiles allows more accurate prediction of motifs than the PSSMs, which are generated mainly based on the position-specific amino acid frequency. The accurate prediction of NESs with the profile-based method argues that many more important protein motifs can be predicted using the same or similar strategies.

## Availability and Future Directions

NESmapper is a multiplatform command-line Perl application with activity-based NES profiles, and licensed under the GNU General Public License version 3.0. The source code, unoptimized and optimized activity-based NES profiles, a sample dataset, and an instruction manual are available at http://sourceforge.net/projects/nesmapper.

We plan to develop a NES/NLS prediction tools by combining NESmapper and the previously developed cNLS Mapper. Because many of NES-containing proteins have also NLSs, the simultaneous prediction of NESs and NLSs should be useful for not only identifying nucleo-cytoplasmic shuttling-proteins but also increasing the prediction accuracy for NESs and NLSs. The combined program will be also provided by a webserver, and possibly integrated with structural information of proteins in the future.

## Supporting Information

Figure S1 **Semiquantitative measurement of NES activity.** (**A**) Two representative phenotypes of GFP localization. The GFP–NES reporter fusion protein in NIH3T3 cells localized evenly to both the nucleus and cytoplasm when the fused NES had no nuclear export activity (NC-phenotype), whereas it localized exclusively to the cytoplasm when it had strong NES activity (C-phenotype). (**B**) Score representation of relative levels of NES activity. The proportion of cells with the C-phenotype increased as the activity of the fused NES increased. NES activity was ranked from 1 to 10 based on the proportion of cells with the GFP C-phenotype among all the GFP-positive cells. The scoring was standardized as follows: score 1 (0%–5% of C-phonotype), 2 (6%–10% of C-phonotype), 3 (11%–20% of C-phonotype), 4 (21%–35% of C-phonotype), 5 (36%–50% of C-phonotype), 6 (51%–60% of C-phonotype), 7 (61%–70% of C-phonotype), 8 (71%–80% of C-phonotype), 9 (81%–90% of C-phonotype), and 10 (91%–100% of C-phonotype). In some cases, the relative difference in the intensity of the GFP fluorescence in the nucleus and the cytoplasm was used to determine the final score. Several scores of <1 and >10 were estimated based on the activities determined with a different template with a contrasting level of basal activity.
(PDF)

Figure S2 **Amino acid composition of sequences flanking positive and negative NESs.** Five-amino-acid flanking sequences of a 14-amino-acid NES, starting at position −25, −20, −15, −10, −5, 15, 20, 25, 30, or 35 (where the first amino acid of the NES is regarded as position 1) were extracted and the contents of the indicated amino acids (**A**,**B**: hydrophobic; **C**,**D**: polar; **E**,**F**: acidic; **G**,**H**: basic; **I**,**J**: proline) were calculated for each positive and negative NES dataset. The positive datasets (blue squares) consisted of 178 NESs from the ValidNES dataset and the negative datasets (red circles) consisted of 1,259 NESs from the ValidNES dataset (**A,C,E,G,I**) and 2,078 NESs from the Sp-protein dataset (**B,D,F,H,J**).
(PDF)

Software S1 **The source code of NESmapper, activity-based NES profiles, instructions, and sample data.**
(GZ) Click here for additional data file.

Table S1 **Datasets used for profile-optimizations and performance-tests in this study.**
(PDF)

Table S2 **Frequency/probability distribution of the hydrophobic-to-polar amino acid ratio in the flanking sequences of positive and negative NESs and the calculated likelihood ratios.**
(PDF)

Table S3 **Frequency/probability distribution of the net charge in the flanking sequences of the positive and negative NESs and the calculated likelihood ratios.**
(PDF)

Table S4 **Improvement of the prediction performance of NESmapper by incorporating the properties of the amino acids composing the NES-flanking sequences.**
(PDF)

Table S5 **Observed frequencies of amino acid at the conserved hydrophobic positions of class 1 NESs in positive and negative datasets.**
(PDF)

Table S6 **Observed and expected frequencies of an amino acid pair at the conserved hydrophobic positions of the class 1 NES in the positive and negative datasets.**
(PDF)

Table S7 **NES prediction for 500 budding yeast proteins.**
(PDF)

Table S8 **Running time of NESmapper and NESsential.**
(PDF)

Text S1 **Detailed description of** **Design and Implementation** **.**
(PDF)
