# Supplementary material for: NESmapper: Accurate Prediction of Leucine-Rich Nuclear Export Signals Using Activity-Based Profiles
Source: PLoS Comput Biol. 2014 Sep 18;10(9):e1003841. doi: 10.1371/journal.pcbi.1003841 (PMC4168985; doi:10.1371/journal.pcbi.1003841)
Supplement: Figure S1 — Semiquantitative measurement of NES activity. (A) Two representative phenotypes of GFP localization. The GFP–NES reporter fusion protein in NIH3T3 cells localized evenly to both the nucleus and cytoplasm when the fused NES had no nuclear export activity (NC-phenotype), whereas it localized exclusively to the cytoplasm when it had strong NES activity (C-phenotype). (B) Score representation of relative levels of NES activity. The proportion of cells with the C-phenotype increased as the activity of the fused NES increased. NES activity was ranked from 1 to 10 based on the proportion of cells with the GFP C-phenotype among all the GFP-positive cells. The scoring was standardized as follows: score 1 (0%–5% of C-phonotype), 2 (6%–10% of C-phonotype), 3 (11%–20% of C-phonotype), 4 (21%–35% of C-phonotype), 5 (36%–50% of C-phonotype), 6 (51%–60% of C-phonotype), 7 (61%–70% of C-phonotype), 8 (71%–80% of C-phonotype), 9 (81%–90% of C-phonotype), and 10 (91%–100% of C-phonotype). In some cases, the relative difference in the intensity of the GFP fluorescence in the nucleus and the cytoplasm was used to determine the final score. Several scores of <1 and >10 were estimated based on the activities determined with a different template with a contrasting level of basal activity. (PDF) [file pcbi.1003841.s001.pdf]

A

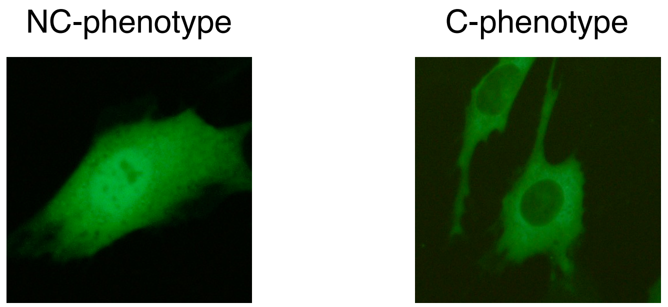

B

Scores	1	2	3	4	5	6	7	8	9	10
Cells with C-phenotype (%)	0~ 5	6~ 10	11~ 20	21~ 35	36~ 50	51~ 60	61~ 70	71~ 80	81~ 90	91~ 100

Figure S1. Semiquantitative measurement of NES activity.
(**A**) Two representative phenotypes of GFP localization. The GFP–NES reporter fusion protein in NIH3T3 cells localized evenly to both the nucleus and cytoplasm when the fused NES had no nuclear export activity (NC-phenotype), whereas it localized exclusively to the cytoplasm when it had strong NES activity (C-phenotype). (**B**) Score representation of relative levels of NES activity. The proportion of cells with the C-phenotype increased as the activity of the fused NES increased. NES activity was ranked from 1 to 10 based on the proportion of cells with the GFP C-phenotype among all the GFP-positive cells. The scoring was standardized as follows: score 1 (0%–5% of C-phonotype), 2 (6%–10% of C-phonotype), 3 (11%–20% of C-phonotype), 4 (21%–35% of C-phonotype), 5 (36%–50% of C-phonotype), 6 (51%–60% of C-phonotype), 7 (61%–70% of C-phonotype), 8 (71%–80% of C-phonotype), 9 (81%–90% of C-phonotype), and 10 (91%–100% of C-phonotype). In some cases, the relative difference in the intensity of the GFP fluorescence in the nucleus and the cytoplasm was used to determine the final score. Several scores of < 1 and > 10 were estimated based on the activities determined with a different template with a contrasting level of basal activity.
