# Supplementary material for: NESmapper: Accurate Prediction of Leucine-Rich Nuclear Export Signals Using Activity-Based Profiles
Source: PLoS Comput Biol. 2014 Sep 18;10(9):e1003841. doi: 10.1371/journal.pcbi.1003841 (PMC4168985; doi:10.1371/journal.pcbi.1003841)
Supplement: Figure S2 — Amino acid composition of sequences flanking positive and negative NESs. Five-amino-acid flanking sequences of a 14-amino-acid NES, starting at position −25, −20, −15, −10, −5, 15, 20, 25, 30, or 35 (where the first amino acid of the NES is regarded as position 1) were extracted and the contents of the indicated amino acids (A,B: hydrophobic; C,D: polar; E,F: acidic; G,H: basic; I,J: proline) were calculated for each positive and negative NES dataset. The positive datasets (blue squares) consisted of 178 NESs from the ValidNES dataset and the negative datasets (red circles) consisted of 1,259 NESs from the ValidNES dataset (A,C,E,G,I) and 2,078 NESs from the Sp-protein dataset (B,D,F,H,J). (PDF) [file pcbi.1003841.s002.pdf]

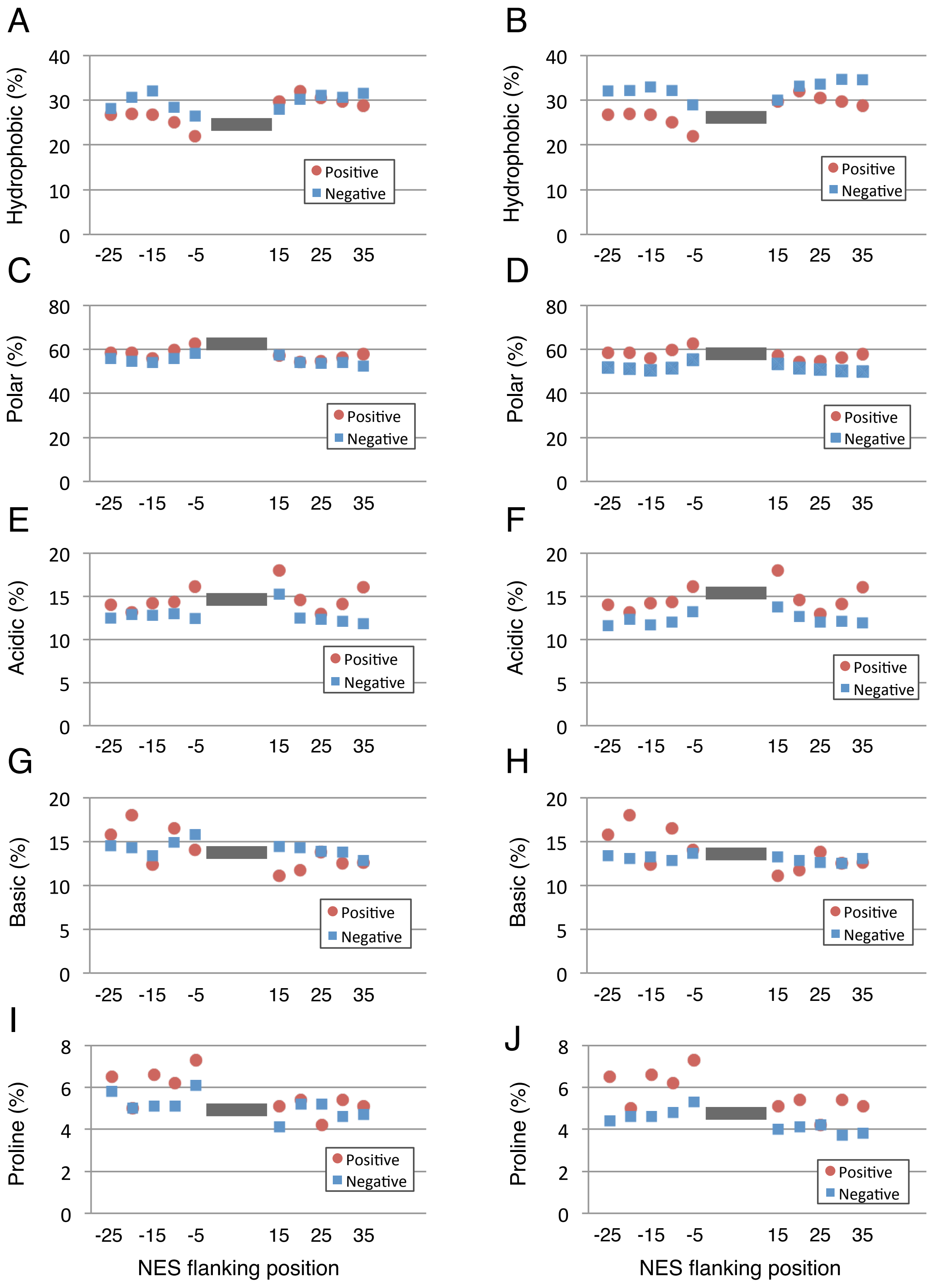

Figure S2. Amino acid composition of sequences flanking positive and negative NESs. Five-amino-acid flanking sequences of a 14-amino-acid NES, starting at position −25, −20, −15, −10, −5, 15, 20, 25, 30, or 35 (where the first amino acid of the NES is regarded as position 1) were extracted and the contents of the indicated amino acids (**A**,**B**: hydrophobic; **C**,**D**: polar; **E**,**F**: acidic; **G**,**H**: basic; **I**,**J**: proline) were calculated for each positive and negative NES dataset. The positive datasets (blue squares) consisted of 178 NESs from the ValidNES dataset and the negative datasets (red circles) consisted of 1,259 NESs from the ValidNES dataset (**A,C,E,G,I**) and 2,078 NESs from the Sp-protein dataset (**B,D,F,H,J**).
