# Supplementary material for: NESmapper: Accurate Prediction of Leucine-Rich Nuclear Export Signals Using Activity-Based Profiles
Source: PLoS Comput Biol. 2014 Sep 18;10(9):e1003841. doi: 10.1371/journal.pcbi.1003841 (PMC4168985; doi:10.1371/journal.pcbi.1003841)
Supplement: Table S1 — Datasets used for profile-optimizations and performance-tests in this study. (PDF) [file pcbi.1003841.s004.pdf]

Table S1. Datasets used for profile-optimizations and performance-tests in this study

(1) ValidNES dataset

(a) Test set (ValidNES-test)		(b) Training set for profiles	
NES ACC	Protein ID (UniProt)	NES ACC	Protein ID (UniProt)
S001	P61925	S004	Q9W639
S002	P04637	S007	P00520
S005	O60716-18	S008	P14635
S006	P49138	S009	P60010
S010	P60010	S013	Q05116
S011	P05865	S014	P42566
S016	P38398	S015	P38398
S017	Q04206	S019	Q01663
S018	P41152	S021	Q14872
S020	P42224	S025	O35973
S024	P13350	S027	P03243
S026	P10688	S029	P47973
S028	O35864	S030	P25963
S032	Q14653	S031	P43487
S034	Q15797	S033	O15350
S037	Q13485	S035	Q9BY84
S039	P46934-4	S036	Q62074
S044	P25054	S038	P14373
S051	O00221	S042	P30309
S052	P54824	S043	P30309
S060	Q91UL8	S047	Q9WTZ9
S073	P38861	S048	Q04377
S077	P56524	S053	O14746
S078	Q16254	S059	O54943
S079	Q16254	S076	Q9UQL6
S085	Q04360	S087	P0CA03
S086	P0CA03	S091	O94916
S088	O00401	S095	P08353
S089	O60729	S097	P15336
S090	O70201	S098	P16110
S092	P03409	S101	P17861
S093	P06748	S103	P20067
S094	P06748	S105	P22363
S096	P11142	S108	P38483
S099	P16727	S109	P42345
S100	P16727	S111	P42345
S102	P19508	S113	P50616
S104	P21952	S116	P97318-2
S106	P28289	S117	P97318-2
S107	P30021	S119	Q14145
S110	P42345	S122	Q16236-2
S112	P42858	S123	Q16236-2
S114	P59636	S125	Q5EBH1
S115	P67775	S127	Q61699
S118	Q02085	S129	Q8IXJ6
S120	Q14494	S130	Q8K3Y6
S121	Q14678	S131	Q8N720
S124	Q18PE1	S132	Q95168
S126	Q60795	S135	Q96JZ2
S128	Q6UB99	S137	Q96T21
S133	Q95168	S138	Q99152
S134	Q95168	S139	Q99MU3
S136	Q96T21	S143	Q9GZX7
S140	Q9BRK4	S144	Q9HC62
S141	Q9EPW1	S150	Q9Y572
S142	Q9EPW1	S151	Q9Z2A0
S145	Q9PWE8	S157	Q99AM3
S146	Q9R1A8	S158	Q9YZN9
S147	Q9UMX3	S161	P51587
S148	Q9UNH5	S162	Q02880
S149	Q9Y572	S164	Q83414
S154	O92398	S166	P05230
S155	Q5GLC3	S169	P53686
S159	P11388	S172	O15265
S160	P11388	S173	O75398
S163	Q68SB1-2	S176	P12272
S165	P02554	S177	P30022
S174	P03322	S178	P34078
S175	P04406	S181	Q09728
S180	P35232	S182	Q96AP0
S183	Q99704	S184	Q9BZL6
S196	O43196	S185	Q9QY16
S198	Q9Y3M2	S186	Q9Y2W2
S201	Q8VIP2	S187	P46527
S202	O15519	S188	Q997F2
S209	O95613	S190	Q9HBL8
S212	P03419	S191	P15336
S213	Q01658	S192	Q86TB9
S214	Q9BZB8	S193	P78545
S216	Q9Z0Z7	S194	P78545
S217	Q16665	S195	Q15172
S220	Q24167	S197	Q01354
S223	P03116	S199	O08560
S224	O15392	S203	P30291
S225	O15392	S204	Q99612
S231	Q14994	S205	O95613
S234	Q14674	S206	O95613
S235	Q8BMI0	S215	Q9BZB8
S236	Q9UDY8	S219	P03372
S239	O42250	S221	Q24167
S246	Q9H9S0	S222	Q14140
S247	Q6NXT1	S228	P36328
S249	P23528	S229	P07355
S250	P15682	S230	O15392-4
S252	Q8NFU5	S232	Q9QUS1
S253	Q9UK80	S238	P31749
S256	Q9WVH5	S240	Q9LMK7
S257	P61023	S244	P03255
S258	P61023	S248	P16800
S260	P0C686	S251	P24097
		S254	Q8N108-13
		S255	P20651
		S259	Q12824

(2) Sp-protein

(a) Test set (Sp-test)	(b) Training set	
Protein ID (Pombase)	Protein ID (Pombase)	
SPCC191.04c	SPAC7D4.07c	SPBC428.02c
SPAC664.13	SPBC21C3.19	SPAC19B12.08
SPCC191.03c	SPBC4B4.12c	SPAC5H10.04
SPBC25H2.09	SPAC1142.09	SPAC17G6.04c
SPAC29B12.11c	SPBC660.09	SPBC12C2.04
SPBC557.02c	SPAC869.09	SPAC5H10.10
SPBP4G3.03	SPCC18B5.09c	SPAC3F10.09
SPCC126.10	SPCC16A11.15c	SPAC24C9.03
SPBC1A4.04	SPAC227.17c	SPCC13B11.04c
SPAC25B8.10	SPAC227.05	SPBC23G7.10c
SPBC839.14c	SPBC19C7.04c	SPCC1223.14
SPBC3E7.07c	SPBC21C3.12c	SPAC25B8.13c
SPBC119.03	SPCC285.04	SPAC2F3.11
SPBC21C3.10c	SPAC2C4.04c	SPAC1002.18
SPAC4H3.08	SPAC3G6.07	SPCC1672.01
SPBC3H7.12	SPBC13G1.06c	SPACUNK4.17
SPCC1235.04c	SPAC11D3.04c	SPCC4G3.16
SPAC2G11.04	SPAC22F8.03c	SPAC1556.08c
SPBC1683.06c	SPCC191.08	SPBC646.06c
SPCC1183.02	SPAC29B12.13	SPAC23H4.16c
SPCC1442.16c	SPBC216.04c	SPAC10F6.13c
SPBC18E5.05c	SPAC6F6.05	SPAC869.04
SPCC1020.07	SPAC22G7.11c	SPCC11E10.07c
SPAC8E11.05c	SPBC19G7.18c	SPBC56F2.06
SPAC1071.05	SPAC29A4.05	SPBC2G2.13c
SPBC25B2.06c	SPBC405.05	SPBC354.15
SPAC23H4.06	SPBC887.08	SPBC1271.04c
SPAC23H3.09c	SPBC146.08c	SPAC3H1.10
SPBC4C3.09	SPAC16E8.03	SPAC1039.06
SPAC15E1.05c	SPAC821.10c	SPCC965.12
SPBC660.12c	SPAC26H5.10c	SPAC6B12.04c
SPCC895.03c	SPBC2D10.03c	SPCC777.08c
SPAC4G8.06c	SPAC57A10.06	SPBC21C3.11
SPAC4F8.07c	SPBC83.12	SPBC8E4.05c
SPBC1539.07c	SPBC31F10.02	SPAC13G7.12c
SPBC2G2.16	SPAC56E4.02c	SPAC15A10.04c
SPCC794.10	SPBC354.04	SPAP8A3.07c
SPAC4F8.14c	SPBC16H5.15	SPAC1F8.04c
SPBP8B7.17c	SPAC1F12.02c	SPBC3B8.05
SPCC757.07c	SPAC1783.06c	SPBC13G1.02
SPAC22F3.10c	SPBC26H8.11c	SPBC1289.08
SPBC21H7.07c	SPCC191.01	SPBC28F2.05c
SPBC8D2.16c	SPCC16C4.16c	SPBC36.06c
SPBC29A3.02c	SPAC227.04	SPCC576.14
SPAC13C5.05c	SPCC1494.09c	SPAC869.02c
SPBC21.02	SPCC417.04	SPAC12G12.11c
SPAC607.06c	SPAC926.03	SPBC428.11
SPAC10F6.03c	SPAC186.06	SPAC821.05
SPAC17A5.15c	SPBC36.10	SPAC630.07c
SPCC1259.11c	SPAC10F6.07c	SPBC3E7.10
SPBC15D4.15	SPAC8C9.16c	SPCC18.10
SPBPB2B2.08	SPAC29E6.05c	SPBC1778.03c
SPAC20H4.05c	SPAC15A10.07	SPACUNK4.10
SPAC7D4.05	SPCC24B10.03	SPBC106.11c
SPAC644.09	SPAC19G12.04	SPBC21C3.08c
SPAC3C7.07c	SPAC4F10.19c	SPCC320.09
SPAC20H4.06c	SPCC576.03c	SPAC21E11.06
SPCPJ732.02c	SPAC4G9.12	SPCC1393.13
SPBPB10D8.02c	SPAC5H10.05c	SPAC24H6.04
SPBCPT2R1.03	SPCC4G3.17	SPCC1840.04
	SPCC4B3.06c	SPAC922.07c
	SPAC25H1.03	SPCC550.10
	SPBP8B7.24c	SPAC806.04c
	SPCC1919.07	SPCC1322.04
	SPBP35G2.02	SPAC1296.03c
	SPACUNK4.15	SPAC8E11.01c
	SPBC215.02	SPAC22H10.08
	SPCC1450.13c	SPAC22A12.16
	SPAC21E11.04	SPCC417.12
	SPAC26F1.06	SPBP8B7.21
	SPAC513.02	SPAC1805.10
	SPAC24H6.08	SPCC285.09c
	SPBC409.17c	SPCC550.07
	SPAC22E12.03c	SPAC9E9.06c
	SPAC22H10.02	SPAC3F10.06c
	SPCC191.09c	SPAC323.06c
	SPAC4F10.20	SPCC14G10.04
	SPAC17A2.10c	SPAC1296.01c
	SPAC869.08	SPCC191.02c
	SPCC965.07c	SPCC23B6.01c
	SPAC644.08	SPBC2G5.05
	SPAC222.08c	SPAC19A8.15
	SPBC23E6.06c	SPCC1840.05c
	SPCC576.02	SPAC9.09
	SPAC1F7.10	SPBC1604.01
	SPCP1E11.10	SPAC3G6.09c
	SPAC5H10.02c	SPCC162.02c
	SPBC530.07c	SPAC3H1.02c
	SPAC31G5.05c	SPCC1494.07
	SPBC26H8.06	SPBC8D2.11
	SPCC757.03c	SPBC336.10c
	SPAC13D6.03c	SPBC32F12.03c
	SPAC17A5.05c	SPBC28F2.03
	SPBC30D10.05c	SPBC36B7.06c
	SPAC13C5.04	SPAC31G5.08
	SPBC30D10.14	SPAC4D7.12c
	SPCC24B10.21	SPBP26C9.02c
	SPAC25B8.09	SPBC36B7.02
	SPAC1F7.06	SPBC14F5.05c
	SPBC839.03c	SPBC215.05
	SPCC24B10.20	SPBC25D12.04
	SPBC359.06	SPBC14F5.04c
	SPBC725.03	SPAC23G3.03

(3) Artificial NESs

(a) Positive dataset		(b) Negative dataset	
NES name	NES sequence	NES name	NES sequence
class-1a-1	EKENTMEALFRRLRLR	class-1a-1	TMTWLIRWNLFRLGLL
class-1a-2	NTMGGFTSSLSRLHID	class-1a-2	NTMRVLQIWLRELCLD
class-1a-3	EKENTMCDIMQNLSLK	class-1a-3*	EKENTMLRTLTLWRLP
class-1a-4	NTMEDLCAMVAVLSVD	class-1a-4*	EKENTMIFPITLCGLR
class-1a-5	ENTMDVVQELAGFSLY	class-1a-5*	TMPSSLAEKLAGWDCN
class-1a-6	ENTMTLGERLYCLMLE	class-1a-6	GGPSSLAEKLAGFDWN
class-1a-7	NTMALVSERLAAASLD	class-1a-7	GGPSSLAEKLAGWDFT
class-1a-8	NTMLPINERLSHLSLD	class-1a-8	GGPSSLADKLAGCDWN
class-1a-9	KENTMLEVALRSLRVC	class-1a-9	GGPSSLAEKLAGWDWN
class-1a-10	ENTMQVESGVSKLVLS	class-1a-10	GGPSSLAEKLAGFDCN
class-1a-11	NTMIDVVIGLAYLTLD	class-1a-11	GGPSSLAEKLAGCDCN
class-1a-12	ENTMGLWADFAELSFA	class-1a-12	GGPSSFAEKLAGLDFN
class-1a-13	EKENTMLTAMDRLYIG	class-1a-13	GGPSSLAEKPAGLDFN
class-1a-14	EKENTMAEMLSSFALT	class-1a-14	GGPSSLAEKLAGLDWN
class-1a-15	NTMSTLSQHLSRLSTD	class-1a-15	GGPSSWAEKLAGLDFN
class-1a-16	NTMDELCRVFCRLYVD	class-1a-16	GGPSSWAEKLAGLDCN
class-1a-17	EKENTMLGSLASLSLE	class-1a-17	GGPSSCAEKLAGLDFN
class-1a-18	ENTMPVIPSLAPLSIR	class-1a-18	GGPSSCAEKLAGRDCN
class-1a-19	TMLFNFEKEITMLSLD	class-1a-19	GGPSSCAEKLAGLDWN
class-1a-20	EKENTMAKDFVSLSLA	class-1a-20	GGPSSVAEKLFTLGIN
class-1a-21	KENTMVASRLAMLMLL	class-1a-21	GGPSSVAEKLTTLPIN
class-1a-22	NTMEALESSINRLCVD	class-1a-22	GGPSSVAEKLFPLNIN
class-1a-23	NTMSIITMDLSALTLD	class-1a-23	GGPSSVAEKLDFLWIN
class-1a-24	KENTMICDLLTRLSCA	class-1a-24	GGPSSVAEKLWVLPIN
class-1a-25	ENTMAVETILSCLAIA	class-1a-25	GGPSSVAEKLTLLPIN
class-1a-26	ENTMGILTDLAKLGVA	class-1a-26	GGPSSVAEKLSLLPIN
class-1a-27	NTMMCLCFGLAGLRLD	class-1a-27	GGPSSVAEKLGPLPIN
class-1a-28	EKENTMVQELNKLELS	class-1a-28	GGPSSVAEKLLPLGIN
class-1a-29	ENTMGLARGVSRLTIT	class-1a-29	GGPSSVAEKLSLLPIN
class-1a-30	NTMTLLANHLGHVTID	class-1a-30	GGPSSVAEKLHVLLIN
class-1a-31	NTMATITEHLRTLYMD	class-1a-31	GGPSSVAEKLAPLPIN
class-1a-32	KENTMCCTLNSLSLVS	class-1a-32	GGPSSVAEKLPNLGIN
class-1a-33	NTMLRTARELAVLRLD	class-1a-33	GGPSSVAEKLRYLPIN
class-1a-34	NTMQRLEVCLSRFAID	class-1b-1*	FEGENTMRVWSQLRTT
class-1a-35	KENTMIGTFLRHLSLS	class-1b-2*	KENTMNVKWLYDMHCN
class-1a-36	EKENTMSRSLAALTLR	class-1b-3*	ENTMERLWTYPGLTCL
class-1a-37	EKENTMTDRLGLLTLS	class-1b-4*	KENTMNLDAFWRLDIG
class-1a-38	KENTMLRSHLGSLSLT	class-1c-1*	SSTMLWQAVGTSFGTL
class-1a-39	EKENTMVQQLRGLSLG	class-1c-2	PSSELAEKLPRRLDLN
class-1a-40	NTMSTMQISFSQLAVD	class-1c-3	PSSELAEKLLQPLDLN
class-1a-41	NTMSRVDRFLQRLTLD	class-1c-4	PSSELAEKLRPLLDLN
class-1a-42	ENTMRLDEAVWKLSLS	class-1c-5	PSSELAEKLAWFLDLN
class-1a-43	NTMCLLSERFIGLRFD	class-1c-6	PSSELAEKLPPVLDLN
class-1a-44	NTMEKMVGLLSACSFD	class-1c-7	PSSELAEKLPLGLDLN
class-1a-45	ENTMRCVELFGSLCLL	class-1c-8	PSSELAEKLAPDLDLN
class-1a-46	NTMDELSTLFTIICLD	class-1c-9	PSSELAEKLPSPLDLN
class-1a-47	EKENTMLPKLRALALT	class-1c-10	PSSELAEKLRPRLDLN
class-1a-48	NTMAGLLTRFFSLAFD	class-1c-11	PSSELAEKLRRPLDLN
class-1a-49	NTMVDVVAGLQGVALD	class-1c-12	PSSELAEKLHPVLDLN
class-1a-50	EKENTMVCKLALLSIS	class-1c-13	PSSELAEKLPTGLDLN
class-1a-51	EKENTMLTTFTNLNLS	class-1c-14	PSSELAEKLAHPLDLN
class-1a-52	NTMRSLISQARSLTLD	class-1c-15	PSSELAEKLRPPLDLN
class-1a-53	KENTMVNMRMARLELS	class-1c-16	PSSELAEKLTAPLDLN
class-1a-54	NTMSQVWHRISQLTLD	class-1c-17	PSSELAEKLPAGLDLN
class-1a-55	NTMGVLSTRLRALGVD	class-1c-18	PSSELAEKLQPGLDLN
class-1a-56	ENTMEVANYLSLLMIN	class-1c-19	PSSELAEPLQAGLDLN
class-1a-57	ENTMDVPISLDGLLLA	class-1c-20	PSSELREKLQAGLDLN
class-1a-58	EKENTMCRQLAALGIR	class-1c-21	PSSELKEKLQAGLDLN
class-1a-59	NTMGVVEERIKRLVFD	class-2-1*	FEGENTMSLPHASVAD
class-1a-60	NTMSGMEERLMALWLD	class-2-2*	KENTRSVLLRSLGAFD
class-1a-61	NTMSQLQMRIEDLVMD		
class-1a-62	ENTMDLAAPLGRLGIG		
class-1a-63	NTMPSVTGALRQLSVD		
class-1a-64	EKENTMCHRFADLTLE		
class-1a-65	ENTMRARAALASLTME		
class-1a-66	NTMSQLQMRIEDLVMD		
class-1a-67	EKENTMDPSTGFLRLC		
class-1a-68	NTMHDIVYGLREVELD		
class-1a-69	EKENTMDVLIERLWAD		
class-1a-70	EKENTMLDAVNGVWLT		
class-1a-71	EKENTVTGALRQLSVD		
class-1a-119	GGPSSCAEKLAGLDMN		
class-1a-119-2	GGPSSLAEKLAGCDLN		
class-1a-166	PSSELLAEKLAGLDLN		
class-1a-113	GGPSSLAEKLAGLDCN		
class-1a-113-2	GGPSSWAEKLAGLDLN		
class-1a-73	GGPSSVAEKLVTLSIN		
class-1a-74	GGPSSVAEKLKSLAIN		
class-1a-75	GGPSSVAEKLNVLSIN		
class-1a-76	GGPSSVAEKLATLGIN		
class-1a-77	GGPSSVAEKLTYLIIN		
class-1a-78	GGPSSVAEKLRYLRIN		
class-1a-79	GGPSSVAEKLGILQIN		
class-1a-80	GGPSSVAEKLQDLKIN		
class-1a-81	GGPSSVAEKLSALLIN		
class-1a-82	GGPSSVAEKLNALHIN		
class-1a-83	GGPSSVAEKLHALCIN		
class-1a-84	GGPSSVAEKLCMLSIN		
class-1a-85	GGPSSVAEKLNTLSIN		
class-1a-86	GGPSSVAEKLIRLAIN		
class-1a-87	GGPSSVAEKLNKLQIN		
class-1a-88	GGPSSVAEKLAGLAIN		
class-1a-89	GGPSSVAEKLMCLKIN		
class-1a-90	GGPSSVAEKLMSLVIN		
class-1a-91	GGPSSVAEKLASLDIN		
class-1a-92	GGPSSVAEKLLTLAIN		
class-1a-93	GGPSSVAEKLVLLHIN		
class-1a-94	GGPSSVAEKLSTLDIN		
class-1a-95	GGPSSVAEKLLCLSIN		
class-1a-96	GGPSSVAEKLGRLDIN		
class-1a-97	GGPSSVAEKLGGLNIN		
class-1a-98	GGPSSVAKKLASLIIN		
class-1a-x1	GGPSSVAEKLHLLAIN		

* NESs from our previous screening study (Kosugi et al., 2008 Traffic 9, 2053-2062)

S261	P40427	SPAC922.06	SPAC1834.01	class-1a-x2	GGPSSVAEKLIGLMIN
S262	P27987	SPCC70.07c	SPBC1711.13	class-1a-x3	GGPSSVAEKLLFLRIN
		SPAC22A12.17c	SPBC1815.01	class-1a-x4	GGPSSVAEKLLWLSIN
		SPCC1739.08c	SPBC4F6.11c	class-1a-x5	GGPSSVAEKLIYLNIN
		SPAC227.14	SPBC32F12.10	class-1a-x6	GGPSSVAEKLGFLRIN
		SPCC330.05c	SPBC119.10	class-1a-x7	GGPSSVAEKLDVLVIN
		SPAC15E1.10	SPCC1450.10c	class-1a-x8	GGPSSVAEKLIYLSIN
		SPBC839.17c	SPAC20G4.05c	class-1b-1	FEGENTMRRFAGLQLE
		SPBC106.15	SPCC16A11.03c	class-1b-2	FEMEQTVAGLFTLTLD
		SPAC16E8.04c	SPAC13A11.06	class-1b-3	FEGENTMDGFGALSLS
		SPAC694.03	SPAC144.03	class-1b-4	FEGENTMMRLCRLMIS
		SPBC17D11.03c	SPBC582.08	class-1b-5	FEGENTMARLVNLAIV
		SPCC965.09	SPAC56F8.10	class-1b-6	FEGENTMRRLRTLVIR
		SPAC17C9.02c	SPBC15D4.09c	class-1b-7	FEGENTMPDMSRLAIR
		SPBC17G9.12c	SPCC1322.05c	class-1b-8	FEGPMNLDAFWRLDIG
		SPAC2F3.05c	SPBP8B7.29	class-1b-9	FEGENTMDQLRALDLN
		SPBC21B10.08c	SPCC1020.13c	class-1b-10	NTMGCALRSMRSLSLD
		SPBC3H7.10	SPBC1703.07	class-1c-1	KENTMSRLLATALKLS
		SPAC1B3.06c	SPBC1861.03	class-1c-2	ENTMLSYGIKHKLSLL
		SPBC14F5.01	SPAP8A3.05	class-1c-3	NTMRLSAAFLKDLTID
		SPCC1442.07c	SPBC23G7.05	class-1c-4	PSSELAEKLSAGLDLN
		SPCC4G3.02	SPCC663.06c	class-1c-5	PSSELAEKLYLMLDLN
		SPAC5H10.08c	SPBC26H8.01	class-1c-6	PSSELAEKLGRGLDLN
		SPAC19G12.09	SPBC19C2.07	class-1c-7	PSSELAEKLGNLLDLN
		SPAC1D4.01	SPBC1A4.02c	class-1c-8	PSSELAEKLNSVLDLN
		SPAC16E8.15	SPAC4D7.05	class-1c-9	PSSELAEKLRRVLDLN
		SPBC3B9.01	SPCC663.10	class-1c-10	PSSELAEKLFVNLDLN
		SPBC337.10c	SPCPB16A4.06c	class-1c-11	PSSELAEKLLRGLDLN
		SPAC23C11.05	SPAC212.02	class-1c-12	PSSELAEKLGEGLDLN
		SPAC3A12.09c	SPAC5H10.03	class-1c-13	PSSELAEKLMTQLDLN
		SPBC106.08c	SPAC977.15	class-1c-14	PSSELAEKLARRLDLN
		SPCC1223.09	SPBPB2B2.05	class-1c-15	PSSELAEKLAWFLDLN
		SPCC16C4.10	SPAC12B10.13	class-1c-16	PSSELAEKLSMVLDLN
		SPBC12C2.07c	SPAP32A8.02	class-1c-17	PSSELAEKLRGQLDLN
		SPAC57A10.03	SPCPB16A4.05c	class-1c-18	PSSELAEKLLRRLDLN
		SPAC5H10.01	SPAPB24D3.06c	class-1c-19	PSSELAEKLQMSLDLN
		SPBC23E6.03c	SPBC3F6.03	class-1c-20	PSSELAEKLEAGLDLN
		SPBC12C2.12c	SPAC26F1.11	class-1c-21	PSSELAEKLHLGLDLN
		SPBC215.10	SPAC29A4.12c	class-1c-22	PSSELAEKLVYMLDLN
		SPAC824.07	SPBC887.01	class-1c-23	PSSELAEKLHAYLDLN
		SPBC3B9.06c	SPAC4H3.06	class-1c-24	PSSELAEKLMLSLDLN
		SPBC215.11c	SPBP4H10.12	class-1c-25	PSSELAEKLRMSLDLN
		SPBC577.08c	SPAC9E9.15	class-1c-26	PSSELAEKLALRLDLN
		SPBC6B1.03c	SPAC9G1.08c	class-1c-27	PSSELAEKLGCALDLN
		SPCC320.06	SPBC216.03	class-1c-28	PSSELAEKLARELDLN
		SPAC57A10.07	SPBC14C8.13	class-1c-29	PSSELAEKLKQELDLN
		SPAC222.11	SPAC18B11.04	class-1c-30	PSSELAEKLRLRLDLN
		SPCC4B3.18	SPAC13G7.06	class-1c-31	PSSELAEKLALGLDLN
		SPAC637.08	SPAC607.04	class-1c-32	PSSELAEKLTKHLDLN
		SPBC2D10.20	SPAC688.03c	class-1c-33	PSSELAEKLYVSLDLN
		SPAC30C2.02	SPBC19G7.02	class-1c-34	PSSELAEKLHNLLDLN
		SPAC15A10.05c	SPAC17A5.13	class-2-1	NTMADATVALRALAID
		SPAC19G12.03	SPBC21D10.08c	class-2-2	NTMKTAQLAFTHLTLD
		SPAC26F1.07	SPAC25B8.12c	class-2-3	ENTMSARYSLEKLSLG
		SPAC4D7.06c	SPAC1687.21	class-2-4	KENTMTTLALHSLDLG
		SPCC1020.06c	SPBC16G5.02c	class-2-5	NTMSLSTLTLNDLNCD
		SPAC3H1.07	SPBC4B4.10c	class-2-6	EKENTMQFGLLSLD
		SPAC1B3.01c	SPAC4H3.14c	class-2-7	FEGENTMLAFRALRLA
		SPAC23H3.15c	SPBC1711.11	class-2-8	FSGENTMQFGLLSLTD
		SPBC8E4.04	SPBC25H2.15	class-2-9	EKENTATVALRALAID
		SPAP27G11.09c	SPCC777.03c		
		SPCC18B5.05c	SPAP27G11.08c		
		SPAC1805.06c	SPAC6F12.13c		
		SPAC24C9.14	SPCC4B3.05c		
		SPCC63.06	SPAC343.14c		
		SPCC63.07	SPAC144.04c		
		SPCC830.10	SPAC139.04c		
		SPAC186.02c	SPAC694.04c		
		SPAC186.07c	SPCC569.07		
		SPAC9E9.11	SPAC56E4.03		
		SPAC823.14	SPBC2D10.05		
		SPAC3A11.11c	SPBC1773.13		
		SPCC1494.01	SPAC13G6.11c		
		SPBC30D10.16	SPAC1F5.07c		
		SPAC513.07	SPBC1198.08		
		SPCC1393.09c	SPAC30C2.08		
		SPACUNK4.09	SPCC1442.09		
		SPCC13B11.03c	SPAC3G9.11c		
		SPAC1F7.12	SPAC186.09		
		SPAC8E11.04c	SPBC418.01c		
		SPAC1039.03	SPBC19C7.08c		
		SPBP4H10.17c	SPBC1711.12		
		SPBP35G2.04c	SPBC24C6.09c		
		SPAC1F7.09c	SPAP27G11.12		
		SPBC18E5.01	SPAC22F8.11		
		SPCC1223.02	SPCC1672.06c		
		SPCC4G3.03	SPAPB24D3.08c		
		SPBC651.02	SPBPB2B2.09c		
		SPCC1753.04	SPCC576.01c		
		SPAC959.04c	SPBC365.20c		
		SPBC16G5.08	SPAC977.14c		
		SPAC2E1P5.04c	SPBPB2B2.11		
		SPCC777.06c	SPBP22H7.06		
		SPAC1F12.08	SPBPB2B2.10c		
		SPAC4H3.04c	SPAC4A8.14		
		SPBC23E6.10c	SPAC3H5.11		
		SPAC922.03	SPBC11B10.02c		
		SPBC18H10.20c	SPBC1348.04		
		SPAC3G9.09c	SPAC977.02		
		SPAC806.06c	SPAC977.08		
		SPAC22E12.04	SPBC32H8.01c		
		SPAC513.06c	SPAC27D7.09c		
		SPBC115.03	SPBC16E9.19		
		SPCC965.08c	SPAP27G11.16		
		SPBC1711.04	SPBC16G5.19		
		SPAC1F12.05	SPBC13G1.15c		
		SPAC6F6.11c	SPAC343.20		
		SPBP4H10.05c	SPAC2F3.12c		
		SPBC1709.16c	SPAC11E3.14		
