# Supplementary material for: NESmapper: Accurate Prediction of Leucine-Rich Nuclear Export Signals Using Activity-Based Profiles
Source: PLoS Comput Biol. 2014 Sep 18;10(9):e1003841. doi: 10.1371/journal.pcbi.1003841 (PMC4168985; doi:10.1371/journal.pcbi.1003841)
Supplement: Table S2 — Frequency/probability distribution of the hydrophobic-to-polar amino acid ratio in the flanking sequences of positive and negative NESs and the calculated likelihood ratios. (PDF) [file pcbi.1003841.s005.pdf]

Table S2. Frequency/probability distribution of the hydrophobic-to-polar amino acid ratio in the flanking sequences of positive and negative NESs and the calculated likelihood ratios.

HPR [a]	Positive NESs [b]		Negative NESs [c]		Negative NESs [d]		Likelihood ratio-1 [e]	Likelihood ratio-2 [f]
	Freq	Prob1	Freq	Prob2	Freq	Prob3		
≦30	36	0.20	152	0.12	74	0.04	1.68	5.68
31 to 40	31	0.17	153	0.12	142	0.07	1.43	2.55
41 to 50	35	0.20	179	0.14	274	0.13	1.38	1.49
51 to 60	22	0.12	149	0.12	264	0.13	1.04	0.97
61 to 80	28	0.16	290	0.23	584	0.28	0.68	0.56
≧81	26	0.15	336	0.27	740	0.36	0.55	0.41

[a] Range of the hydrophobic-to-polar amino acid ratios (HPRs) in the flanking sequences of positive or negative NESs. The frequency (Freq) and probability (Prob1-Prob3: rate of frequency) of each range were calculated.

[b] Positive NES set consisting of 178 NESs from the ValidNES dataset.

[c] Negative NES set consisting of 1,259 NESs from the ValidNES dataset.

[d] Negative NES set consisting of 2,078 NESs from the Sp-protein dataset.

[e] Likelihood ratio-1: ratio of Prob1 of the positive NESs to Prob2 of the negative NESs from the ValidNES dataset.

[f] Likelihood ratio-2: ratio of Prob1 of the positive NESs to Prob3 of the negative NESs from the Sp-protein dataset.
