# Supplementary material for: NESmapper: Accurate Prediction of Leucine-Rich Nuclear Export Signals Using Activity-Based Profiles
Source: PLoS Comput Biol. 2014 Sep 18;10(9):e1003841. doi: 10.1371/journal.pcbi.1003841 (PMC4168985; doi:10.1371/journal.pcbi.1003841)
Supplement: Table S3 — Frequency/probability distribution of the net charge in the flanking sequences of the positive and negative NESs and the calculated likelihood ratios. (PDF) [file pcbi.1003841.s006.pdf]

Table S3. Frequency/probability distribution of the net charge in the flanking sequences of the positive and negative NESs and the calculated likelihood ratios.

Net charge [a]	Positive NESs [b]		Negative NESs [c]		Negative NESs [d]		Likelihood ratio-1 [e]	Likelihood ratio-2 [f]
	Freq	Prob1	Freq	Prob2	Freq	Prob3		
≦-4	26	0.15	102	0.08	145	0.07	1.80	2.09
-3.9 to -2	29	0.16	179	0.14	361	0.17	1.15	0.94
-1.9 to 0	55	0.31	351	0.28	683	0.33	1.11	0.94
≧0.1	68	0.38	627	0.50	889	0.43	0.77	0.89

[a] Range of amino acid net charge (NC) in the flanking sequences of the positive or negative NESs. The frequency (Freq) and probability (Prob1-Prob3: rate of frequency) for each range were calculated.

[b] Positive NES set consisting of 178 NESs from the ValidNES dataset.

[c] Negative NES set consisting of 1,259 NESs from the ValidNES dataset.

[d] Negative NES set consisting of 2,078 NESs from the Sp-protein dataset.

[e] Likelihood ratio-1: ratio of Prob1 of the positive NESs to Prob2 of the negative NESs from the ValidNES dataset.

[f] Likelihood ratio-1: ratio of Prob1 of the positive NESs to Prob3 of the negative NESs from the Sp-protein dataset.
