# Supplementary material for: NESmapper: Accurate Prediction of Leucine-Rich Nuclear Export Signals Using Activity-Based Profiles
Source: PLoS Comput Biol. 2014 Sep 18;10(9):e1003841. doi: 10.1371/journal.pcbi.1003841 (PMC4168985; doi:10.1371/journal.pcbi.1003841)
Supplement: Table S4 — Improvement of the prediction performance of NESmapper by incorporating the properties of the amino acids composing the NES-flanking sequences. (PDF) [file pcbi.1003841.s007.pdf]

Table S4. Improvement of the prediction performance of NESmapper by incorporating the properties of the amino acids composing the NES-flanking sequences.

Flanking property	NESmapper threshold score	ValidNES data [a]		Sp-protein data [b]
		False negatives (Sensitivity)	False positives	False positives
−	1	44 (0.785)	1,033	2,059
+		47 (0.771)	970	1,931
−	2	53 (0.742)	788	1,655
+		51 (0.751)	850	1,646
−	3	63 (0.693)	618	1,298
+		55 (0.752)	738	1,383

[a] ValidNES dataset consisting of proteins containing 205 LMB-sensitive NESs.

[b] Sp-protein dataset consisting of 484 proteins from *S. pombe* whose subcellular localizations are not affected by LMB-treatment.

The prediction was performed with the untrained NES profiles.
