# Supplementary material for: NESmapper: Accurate Prediction of Leucine-Rich Nuclear Export Signals Using Activity-Based Profiles
Source: PLoS Comput Biol. 2014 Sep 18;10(9):e1003841. doi: 10.1371/journal.pcbi.1003841 (PMC4168985; doi:10.1371/journal.pcbi.1003841)
Supplement: Table S5 — Observed frequencies of amino acid at the conserved hydrophobic positions of class 1 NESs in positive and negative datasets. (PDF) [file pcbi.1003841.s008.pdf]

Table S5. Observed frequencies of amino acid at the conserved hydrophobic positions of class 1 NESs in positive and negative datasets.

Hydrophobic position	Amino acid	Frequency of occurrence (%)	
		Positive NES set	Negative NES set
Φ0	V	15.2 (18)	7.3
	L	12.7 (15)	11.2
	I	10.1 (12)	6.6
	A	7.6 (9)	7.2
	F	6.7 (8)	5.1
	M	3.3 (4)	2.9
	T	1.6 (2)	5.2
Φ1	L	50.0 (114)	25.7
	V	17.5 (40)	16.9
	I	13.6 (31)	14.8
	M	7.0 (16)	5.8
	F	4.8 (11)	11.5
	T	2.2 (5)	8.7
	A	1.3 (3)	11.1
Φ2	L	49.6 (126)	26.5
	F	13.7 (35)	11.4
	I	9.8 (25)	14.6
	V	7.4 (19)	16.4
	M	3.5 (9)	6.1
	A	1.9 (5)	10.9
	T	0.7 (2)	8.3
Φ3	L	72.8 (185)	35.0
	I	9.8 (25)	18.6
	V	7.0 (18)	22.2
	M	4.7 (12)	8.6
	F	1.9 (5)	15.3
	T	1.1 (3)	0
	A	0.7 (2)	0
Φ4	L	54.3 (138)	33.6
	I	20.0 (51)	19.1
	V	11.8 (30)	23.2
	F	4.7 (12)	15.4
	M	3.9 (10)	8.6
	A	1.9 (5)	0
	T	1.5 (4)	0

The indicated percentages are amino acid frequencies at the conserved hydrophobic positions of the class 1 NESs for both positive and negative NES sets. The positive set consisted of a total of 253 NES sequences (170 from the ValidNES database and 83 from the screened artificial NES library) that matched NES consensus sequence of the classes 1a, 1b, and 1c. The negative NES set consisted of 4,142,179 sequences that matched the class 1a consensus sequence ((Φ0)–X2–Φ1–X3–Φ2–X2–Φ3–X–Φ4, where X represents L, I, V, F, or M, but A, T, and C are allowed at either the Φ1 or Φ2 positions) in 962,317 proteins from the invertebrate and plant RefSeq databases. The amino acid frequency at the Φ0 position was also determined for the class 1a NESs. The artificial NESs of the positive dataset were not used for the calculation at the Φ0 position or for counting the Met residues at the Φ1 position because many of the N-terminal residues of the artificial NESs were derived from part of the vector sequence used for library screening. The number in parentheses indicates the number of the corresponding sequence.
