# Supplementary material for: NESmapper: Accurate Prediction of Leucine-Rich Nuclear Export Signals Using Activity-Based Profiles
Source: PLoS Comput Biol. 2014 Sep 18;10(9):e1003841. doi: 10.1371/journal.pcbi.1003841 (PMC4168985; doi:10.1371/journal.pcbi.1003841)
Supplement: Table S6 — Observed and expected frequencies of an amino acid pair at the conserved hydrophobic positions of the class 1 NES in the positive and negative datasets. (PDF) [file pcbi.1003841.s009.pdf]

Table S6. Observed and expected frequencies of an amino acid pair at the conserved hydrophobic positions of the class 1 NES in the positive and negative datasets.

Hydrophobic positions	Amino acid pair	Frequency of occurrence (%)			
		Positive NES set		Negative NES set	
		Observed	Expected	Observed	Expected
Φ1-Φ2	L-L	20.8 (53)	24.8	6.2	6.8
	V-L	9.8 (25)	8.7	3.8	4.4
	**I-L**	**8.2 (21)**	**5.0**	3.4	3.9
	L-F	7.8 (20)	6.9	2.6	2.9
	L-I	5.9 (15)	4.9	3.3	3.7
	**L-V**	**5.9 (15)**	**3.7**	3.6	4.2
Φ1-Φ3	L-L	31.4 (80)	36.4	9.3	9.0
	V-L	11.8 (30)	12.7	5.8	5.9
	I-L	9.0 (23)	9.9	5.0	5.2
	L-I	4.7 (12)	4.9	4.6	4.8
	L-M	3.5 (9)	2.4	2.1	2.2
	F-L	3.1 (8)	3.5	3.9	4.0
	L-V	2.3 (6)	3.5	5.6	5.7
	I-I	1.9 (5)	1.3	3.0	2.7
Φ1-Φ4	L-L	22.0 (56)	27.2	8.8	8.6
	L-I	9.8 (25)	10.0	4.9	4.9
	I-L	8.2 (21)	7.4	4.8	4.9
	V-L	8.2 (21)	9.5	5.6	5.6
	L-V	4.7 (12)	5.9	5.9	5.9
Φ2-Φ3	L-L	40.1 (102)	36.1	9.9	9.3
	F-L	12.9 (33)	10.0	3.9	4.0
	I-L	7.8 (20)	7.1	5.0	5.1
	**L-V**	**4.7 (12)**	**3.5**	5.5	5.9
	V-L	4.7 (12)	5.4	5.4	5.7
	**M-L**	**4.1 (11)**	**2.5**	2.1	2.1
	L-I	3.9 (10)	4.8	4.7	4.9
Φ2-Φ4	L-L	31.4 (80)	26.9	9.2	8.9
	L-I	10.2 (26)	9.9	4.9	5.0
	F-L	8.6 (22)	7.4	3.7	3.8
	L-V	6.6 (17)	5.8	6.0	6.1
	I-L	4.7 (12)	5.3	4.8	4.9
	**F-I**	**3.9 (10)**	**2.7**	2.2	2.1
	V-L	3.9 (10)	4.0	5.4	5.5

Φ3-Φ4	L-L	41.3 (105)	39.5	12.3	11.8
	L-I	12.9 (33)	14.6	6.6	6.7
	L-V	7.8 (20)	8.6	8.0	8.1
	I-L	4.7 (12)	5.3	6.0	6.2
	V-L	3.9 (10)	3.8	7.3	7.4

The indicated percentages are the observed and expected frequencies of amino acids that simultaneously appear at the two hydrophobic positions indicated in the leftmost column. The positive and negative datasets used were the same as those in Table S5. The number in parentheses indicates the number of the corresponding sequence, and only sequence pairs supported by at least ten NESs are represented. The expected frequency was calculated by multiplying the frequencies of the corresponding amino acids and positions, indicated in Table S5. Sequence pairs for which the ratio of the observed and expected frequencies for the positive dataset was less than 0.77 or greater than 1.3 are highlighted in bold. Pairs involving the Φ0 position of class 1a are not represented because only a small number of NESs can be used for the calculation.
