# Supplementary material for: NESmapper: Accurate Prediction of Leucine-Rich Nuclear Export Signals Using Activity-Based Profiles
Source: PLoS Comput Biol. 2014 Sep 18;10(9):e1003841. doi: 10.1371/journal.pcbi.1003841 (PMC4168985; doi:10.1371/journal.pcbi.1003841)
Supplement: Table S7 — NES prediction for 500 budding yeast proteins. (PDF) [file pcbi.1003841.s010.pdf]

Table S7. NES prediction for 500 budding yeast proteins.

Prediction method	Number of NESs predicted	Percentage of proteins containing predicted NESs
Traditional consensus	2,941	93
Improved consensus	4,039	96
Wregex [a]	5,268	98
NESsential [b]	2,985	96
NESmapper [c]	801	69

The input 500 proteins were randomly selected from the *Saccharomyces cerevisiae* protein database.

[a] Prediction with relaxed PSSM configuration.

[b] NESs with the probability values of ≥0.1 selected.

[c] Prediction with optimized NES profiles. NESs with a score of ≥4 were selected.
