# Supplementary material for: NESmapper: Accurate Prediction of Leucine-Rich Nuclear Export Signals Using Activity-Based Profiles
Source: PLoS Comput Biol. 2014 Sep 18;10(9):e1003841. doi: 10.1371/journal.pcbi.1003841 (PMC4168985; doi:10.1371/journal.pcbi.1003841)
Supplement: Table S8 — Running time of NESmapper and NESsential. (PDF) [file pcbi.1003841.s011.pdf]

Table S8. Running time of NESmapper and NESsential.

Tools		Running time
NESmapper		8 seconds
NESsential [a]	1st step (SABLE)	301 minutes
	2nd step (POODLE)	102 minutes
	Total	403 minutes

The dataset used contains 200 proteins with each 800 amino acids.

[a] NESsential has two steps of the sequential processes, accompanying the implementation of SABLE and POODLE-L.
