# Supplementary material for: NESmapper: Accurate Prediction of Leucine-Rich Nuclear Export Signals Using Activity-Based Profiles
Source: PLoS Comput Biol. 2014 Sep 18;10(9):e1003841. doi: 10.1371/journal.pcbi.1003841 (PMC4168985; doi:10.1371/journal.pcbi.1003841)
Supplement: Text S1 — Detailed description of Design and Implementation . (PDF) [file pcbi.1003841.s012.pdf]

Text S1. Detailed description of Design and Implementation

NES data sets

[1] Positive datasets: We used three positive NES sets, consisting of 205 NESs from the ValidNES database (ValidNES dataset), 32 experimentally verified NESs from the human deubiquitinase family (DUB NESs), and 311 artificial NESs from our studies (positive artificial NES dataset). The DUB NESs were used only for training of NES profiles. The artificial NESs were derived from 93 NESs obtained in a previous study by library screening, and from 70 NESs with double or triple mutations (see Figure 1) and 148 NESs with a single mutation, which were used for the generation of NES profiles, from this study. The ValidNES set was obtained by subtracting 15 NESs with potentially weak activity or no experimental evidence, which were present in multiple NES-containing proteins, from 220 LMB-sensitive NESs.

[2] Negative datasets: The four negative datasets consisted of 1,607 potentially nonfunctional NESs predicted from 424 LMB-unaffected fission yeast proteins (Sp-proteins), 853 potentially nonfunctional NESs predicted from regions other than confirmed NES positions in the positive ValidNES dataset, 78 experimentally verified NESs from the human deubiquitinase family (DUB NESs), and 177 artificial NESs from our studies (negative artificial NES dataset). The artificial negative NESs were derived from 10 NESs in a previous screening study, and 50 NESs with double or triple mutations (see Figure 1) and 117 NESs with a single mutation, which were used for the generation of NES profiles, from this study. To balance the negative and positive training sets for the artificial NESs, we added 274 virtual negative NESs to the negative dataset that have a nonhydrophobic residue at a conserved hydrophobic position. To obtain fission yeast proteins whose subcellular localizations were not affected by LMB treatment, we selected proteins from the *S. pombe* postgenome database (http://www.riken.jp/SPD/) with the annotation 'no change' for the effect of LMB and that contained with $\geq$ 100 amino acids. Selection was also restricted to those proteins with cytoplasmic or partially nuclear subcellular localization because it is difficult to observe changes in subcellular localization in proteins that localize exclusively to the nucleus. We also

selected only those proteins whose functions are unrelated to proteins that function in the nucleus because most NES-containing proteins are involved in nuclear functions. The potentially nonfunctional NESs in the Sp-protein dataset were predicted by selecting NESs with scores of ≥2 using NESmapper with unoptimized profiles. Similarly, potentially nonfunctional NESs in the ValidNES dataset were predicted from regions other than verified NES regions using NESmapper, although we could not exclude the possibility that this dataset contained a number of functional NESs that are yet to be defined. For the preparation of the negative test datasets from the Sp-protein and the ValidNES datasets, the sequences that matched the traditional NES consensus sequences were selected, and all overlapped sequences were removed to generate non-redundant datasets. The 'negative' NES is defined as the sequences that contain the consensus NES sequences but have no nuclear export function. All the sequences of the negative datasets contain the consensus NES patterns of the conserved hydrophobic residues. The nuclear export activities of the negative NESs from the artificial NES dataset have been experimentally confirmed. On the other hand, the negative NESs from the ValidNES and the Sp-protein datasets were selected by prediction, and there is no evidence that these sequences have no NES function. The definition of the negative NESs from the Sp-protein and the ValidNES datasets is based on the assumption that the Sp-proteins and the ValidNES-proteins have no functional LMB-sensitive NESs, except for the defined NES regions.

The training datasets used for profile optimization were prepared by subtracting the test datasets to be used for performance evaluation from both the positive and negative datasets. The complete positive and negative sets for training (train-all sets), including all the test datasets except the Sp-protein test set, were used for training profiles in some cases. These datasets are listed in Table S1. The constitution of the training and test datasets is schematically represented in Figure 2.

For analyzing the amino acid dependency between the conserved hydrophobic residues of NESs, we collected a positive 253 NESs corresponding to classes 1a, 1b, and 1c from the ValidNES database and the artificial NES dataset. To prepare a negative NES set, we selected 4,142,179 sequences that matched the consensus sequence of class 1a from the invertebrate and plant RefSeq protein

databases (a total of 962,317 proteins). Estimating the number of CRM1 cargos to be (at most) 10% of all the proteins, this negative set was likely to contain only a negligible number of functional NESs.

Optimization of NES profiles by training

To allow the faithful calculation of the NES activities, the scores in the NES profiles were optimized to fit the calculation for NESmapper by computational training with positive and negative NES training datasets. NESs with a score greater than –5 for each class from positive and negative datasets were stored and used as the positive and negative NES selection sets for the calculation of the false negative rate (FNR) and the false positive rate (FPR), respectively. FNR was calculated by dividing the number of NESs with a score equal to or smaller than a specified threshold score (2 or 3) by the number of NESs contained in the positive dataset. FPR was determined by dividing the number of NESs with a score greater than or equal to a value one point lower than the threshold score in the negative NES selection set by the number of NESs contained in the negative NES selection set. After the initial determination of FNR and FPR, every score corresponding to a position amino acid in a profile was increased or decreased by 0.5 points and both the FNR and FPR for the corresponding NES class were recalculated. When the sum of FNR and FPR for the NES class was smaller than the prior value, the incrementation or decrementation of the score was continued, and all the scores in all the classes of profiles were adjusted. These procedures were repeated as long as the sum of FNR and FPR was smaller than the value of the previous round. This repetition was usually completed within 10 rounds. Because the efficacy of the score refinement process varied depending on the order of the scores to be adjusted, the entire procedure described above was conducted 200 times and the profiles generated at a procedure giving the lowest sums of FNP and FPR were selected. These procedures were implemented with an in-house Perl script.

Evaluation of NES prediction accuracy

Different positive and negative NES test sets were used to evaluate the prediction of NESs by NESmapper, NESsential, Wregex, NetNES, and NES consensus sequences. NES prediction with consensus sequences was performed with two different types of the consensus sequence: the traditional consensus sequence (Φ–X2,3–Φ-X2,3–Φ–X–Φ) and the improved consensus sequences from our previous study [ref. 14 in the main reference list], modified by Xu et al. [ref. 15 in the main reference list] (i.e., A, T, C, and W are permitted only at the Φ1 and Φ2 positions). The number of NESs with a score smaller than a specified threshold score (2 or 4) were counted as false negatives for the positive data and the number of NESs with a score greater than or equal to the threshold score were counted as false positives for the negative data. For the positive test datasets of the artificial NES and ValidNES datasets, sensitivity was calculated by dividing the number of true positives by the total number of the positive NES sequences. For the negative test dataset of the artificial NESs, specificity was calculated by dividing the number of true negatives by the total number of the negative NES sequences.

For NESsential, the sequential jobs accompanying SABLE, LIBSVM, and POODLE were performed with the program provided (http://seq.cbrc.jp/NESsential/). The sequences with a probability of ≥0.1 in the predicted ordered or disordered region were counted as true NESs unless otherwise stated. For NetNES and Wregex, the query sequences were analyzed on the webservers (http://www.cbs.dtu.dk/services/NetNES/ and http://wregex.ehubio.es, respectively), and the prediction was judged to be true when the predicted NES positions overlapped those of the query sequences. In the predictions of both NESsential and NetNES, a short NES sequence was fused to the C-terminus of the GFP sequence to mimic the NES as part of a protein sequence but the prediction result was evaluated only for the NES portion. In all cases, overlapping NESs were counted as a single predicted NES.
